# Modeling circadian regulation of ovulation timing: age-related disruption of estrous cyclicity

**DOI:** 10.1038/s41598-020-73669-x

**Published:** 2020-10-07

**Authors:** Takayuki Ohara, Takahiro J. Nakamura, Wataru Nakamura, Isao T. Tokuda

**Affiliations:** 1 Institute of Genetics and Biometry, Leibniz Institute for Farm Animal Biology, Dummerstorf, Germany; 2 Laboratory of Animal Physiology, School of Agriculture, Meiji University, Tokyo, Japan; 3 Department of Oral-Chrono Physiology, Graduate School of Biomedical Sciences, Nagasaki University, Nagasaki, Japan; 4 Department of Mechanical Engineering, Ritsumeikan University, Kyoto, Japan

**Keywords:** Oscillators, Ageing, Computational models

## Abstract

The circadian clocks within the hypothalamic–pituitary–gonadal axis control estrous cycles in female rodents. The suprachiasmatic nucleus (SCN), where the central clock is located, generates daily signals to trigger surge release of luteinizing hormone (LH), which in turn induces ovulation. It has been observed in aged rodents that output from the SCN such as neuronal firing activity is declined, and estrous cycles become irregular and finally stop. Circadian clock mutants display accelerated reproductive aging, suggesting the complicated interplay between the circadian system and the endocrine system. To investigate such circadian regulation of estrous cycles, we construct a mathematical model that describes dynamics of key hormones such as LH and of circadian clocks in the SCN and in the ovary, and simulate estrous cycles for various parameter values. Our simulation results demonstrate that reduction of the amplitude of the SCN signal, which is a symptom of aging, makes estrous cycles irregular. We also show that variation in the phase of the SCN signal and changes in the period of ovarian circadian clocks exacerbates the aging effect on estrous cyclicity. Our study suggests that misalignment between the SCN and ovarian circadian oscillations is one of the primary causes of the irregular estrous cycles.

## Introduction

The circadian clock provides an endogenous timekeeping mechanism that generates rhythmicity with a period of about 24 h in many physiological and behavioral processes. While the molecular clocks are present in most organs and tissues in mammals, the suprachiasmatic nucleus (SCN) in the hypothalamus acts as the master circadian pacemaker and coordinates the phase of oscillators in peripheral tissues^1^. A set of core components, i.e. *Period* (*Per*) *1/2*, *Cryptochrome* (*Cry*) *1/2*, *Clock*, and *Bmal1*, as well as *Rev-erb α/β* and *Ror α/β/γ*, constitutes transcriptional/translational feedback loops of the molecular circadian oscillator^2^.

Circadian oscillators within the hypothalamic–pituitary–gonadal (HPG) axis play major roles in the regulation of female reproductive rhythms^3,4^. The importance of the SCN has been demonstrated by experimental observations that a complete lesion of the SCN in rats abolishes a surge of luteinizing hormone (LH) and halts ovulation^5,6^. Two SCN-derived polypeptides, vasopressin (AVP) and vasoactive intestinal peptide (VIP), appear to signal the timing information to gonadotropin-releasing hormone (GnRH) expressing neurons. AVP positive cells in particular target neurons expressing kisspeptin, a strong activator of GnRH release^7,8^. Kisspeptin neurons also express estrogen receptor α^9,10^ and, therefore, they have been considered a good candidate for an integrator of the circadian information and feedback effects of estradiol. Moreover, diurnal and circadian rhythms in FOS expression have been observed in these neurons with the strong activation around the timing of the LH surge^8,11^, suggesting the possibility of rhythmic release of kisspeptin. Kisspeptin signaling is further gated by time-dependent sensitivity to this peptide of GnRH neurons^8^, which may potentially be governed by circadian clocks residing in there^12^. Circadian oscillators present in the ovary^13–15^ also contribute to ovulation timing. Injection of equine LH into rats^16^ and mice^17^ whose endogenous LH release is repressed has revealed a circadian rhythm of oocyte release, suggesting that the ovarian clocks drive a rhythmic sensitivity of preovulatory follicles to LH. Although a circadian rhythm of the *Per1* bioluminescence in cultured granulosa cells gradually attenuates, which is partly due to varied phases of individual cellular clocks, application of LH amplifies the rhythm^18^. Moreover, LH shifts the phase of the *Per1* rhythm in a circadian-time-dependent manner, yielding a phase response curve (PRC) to LH^18^. These experimental observations suggest that LH signals entrain individual ovarian clocks and maintain their synchronization, which might be required to establish the high-amplitude circadian oscillation of the ovary as a whole and to maintain the rhythmic ovulatory sensitivity to LH.

Both the estrous cycle and the circadian system undergo age-related alterations. The estrous cycle is lengthened and becomes irregular with age, which is finally followed by acyclicity^19–21^. The LH surge of aged rodents has been characterized by the delayed onset and the diminished magnitude^22,23^. Aging effects on the circadian system are evident in neural activity of the SCN and subparaventricular zone (SPZ), both of which are essential for circadian output from the SCN^24^. Electrical firing in the SCN and SPZ displays clear diurnal and circadian rhythms in freely moving young mice. Amplitude of the neural activity rhythms is significantly lowered in middle-aged mice, although the accumulated neural activity per day tends to increase^24^. Mechanisms of the decline of the neural activity rhythms in the SCN of old animals have been thoroughly investigated in vitro using male mice^25,26^. Given that clock-gene expressions in the SCN of young and aged rodents show only minor differences under normal light conditions^24,27–29^, it is very likely that the SCN output system, rather than the circadian oscillator itself, is weakened in the aged rodents. This concept is also consistent with the observation that the rhythmicity in mRNA levels of VIP, one of major peptides acting as the SCN output, is attenuated with age^30^.

Impairment of the circadian system exacerbates aging effects on reproductive rhythms. Although mutations of circadian clock genes such as *Per1*, *Per2*, *Cry1* or *Cry2* do not largely affect the regularity of estrous cycles in young adult (2–6 months old) mice, significantly larger proportions of the middle-aged (around 10 months old) mutants are characterized by the irregular or prolonged cycles compared with the wild type of the same age^31,32^. Since irregular estrous cycles observed in the middle-aged mutants generally become evident only at older ages in wild-type rodents^19–21^, it has been concluded that the loss of intact circadian systems accelerates aging process of the reproductive function^31,32^.

Since the circadian system does not only suffer from the age-related changes but also affects itself on an age-related decline of estrous cyclicity, aging effects on the endocrine system and the circadian system need to be investigated together for a comprehensive understanding of aging in the reproductive system. A computational approach using mathematical modeling is useful to investigate and predict the dynamics of complicated biological systems. Although several mathematical models have been proposed for the ovine and bovine estrous cycle and the human menstrual cycle^33–35^, none of them takes the circadian regulation of ovulation timing into account. The purposes of the current study are to develop a mathematical model for the dynamics of the circadian-system-regulated estrous cycle of rodents and to examine how variations in parameters involved with hormone production and with circadian phenotypes impact the estrous cyclicity. We finally propose a possible scenario of how aging makes estrous cycles irregular and how altered circadian rhythmicity results in an accelerated aging. Because of the importance of LH to ovulation, our modeling focuses on two aspects of mutual regulation between LH and the circadian system: (1) timing of the LH surge is controlled by the circadian signal originating from the SCN, and (2) LH acts as a synchronizing agent for ovarian circadian oscillators that drive rhythmic ovulatory sensitivity to LH. We demonstrate that parameter variations causing the attenuation of the LH surge, which is a symptom of the reproductive aging^22,23^, give rise to desynchronization of the ovarian cellular oscillators and a decline of the ovarian sensitivity to LH, resulting in irregular and prolonged estrous cycles. We also show that accelerated aging in circadian clock mutants is at least partly due to misalignment between the SCN and ovarian circadian oscillations, which further reduces synchrony of a population of ovarian cellular oscillators. We thus conclude that the decline of the neural output, which originates from the SCN and is transduced to the ovary as a form of LH surge, causes disorganization of the ovarian circadian system, leading to the irregular estrous cycles.

## Results

### A mathematical model of circadian-clock-regulated ovulation

**Figure 1:**
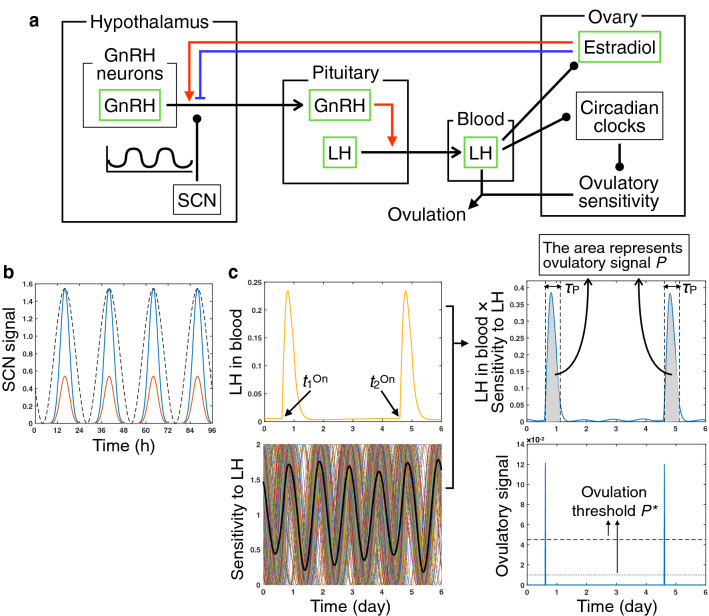
Model describing ovulation dynamics in rodents. (**a**) Schematic representation of the model. GnRH is released from the hypothalamus under the influence of the SCN-derived circadian signal and the estradiol positive (red) or negative (blue) feedback. GnRH in the pituitary then activates LH release. LH in the blood regulates estradiol dynamics and also shifts the phase of ovarian circadian clocks. Ovarian clocks drive rhythmic sensitivity to LH that controls the occurrence of ovulation. (**b**) SCN signal with large (blue) and small (orange) amplitudes. Sine curve (black dashed line) is also represented to emphasize the sharpness of the SCN signal. (**c**) Definition of ovulation in the model (see also Eq. (10)). Ovulatory signal *P* (right panels) is dependent on a product of the LH level in the blood and ovulatory sensitivity to LH (left panels) and, when *P* reaches a threshold P^*^, ovulation occurs. Both high and low values of P^*^ are used in simulations (dashed and dotted lines in a right lower panel, respectively). *t*_*i*_^On^ represents onset time of the *i*th LH surge, and *τ*_P_ represents a duration, over which *P* is calculated. In a left lower panel, rhythmic ovulatory sensitivity to LH is represented for a whole ovary (black thick line) and for individual ovarian cells (other colored solid lines). Heterogeneity of the ovarian circadian oscillators stems from variation of their periods, and the sensitivity of the whole ovary is defined as the mean of the individual sensitivities.

To investigate the occurrence of irregular estrous cycles in aged rodents with putting emphasis on the regulation by the circadian system, we construct a mathematical model consisting of the following four compartments: Hypothalamus, pituitary, peripheral blood, and ovary (Fig. 1a; see “Methods” for detailed explanation). GnRH is synthesized in the hypothalamus and released into the pituitary. The GnRH release is modulated by the SCN-derived circadian signal (Fig. 1b) and is dependent on feedback of estradiol which is produced in the ovary. LH is synthesized in the pituitary and its release into the blood is up-regulated depending on GnRH levels. LH then influences the estradiol dynamics in the ovary so that estradiol sharply decreases after the LH surge (hereafter referred to as LS and defined as an event in which LH in the blood exceeds the threshold L^*^). LH induces phase shifts and synchronizes a population of heterogeneous ovarian circadian oscillators, the periods of which are assumed to be normally distributed. The circadian oscillator drives phase-dependent responsiveness of individual ovarian cells to LH. Ovulation occurs when the ovulatory signal *P*, which depends upon a product of a LH surge level and the whole ovary’s responsiveness to LH (defined as the mean sensitivity of individual cells), exceeds the ovulation threshold P^*^ (Fig. 1c).

It has been known that GnRH, LH, follicle stimulating hormone (FSH), progesterone, and estradiol are the major hormones involved in the ovulation. Among them, both GnRH and LH, which have been clearly shown to interact with the circadian system, are included into our model. Estradiol is also chosen as the main model component because of the essential role of its feedback in the induction of the LH surge.

In the following, we first show the dynamics of each hormone in the default state, where regular 4-day estrous cycles are observed. We then examine how smaller or larger values of the model parameters, such as the amplitude of the SCN signal, affect the estrous cyclicity and demonstrate that decreased parameter values result in irregular cycles. The irregularity is also observed in simulations, where phase of the SCN-signal is weekly and repeatedly perturbed. Such condition is intended to realize the social jetlag^36,37^ as well as the experiments performed by Takasu et al. (2015)^32^. We finally examine impact of the mutation of the ovarian circadian clock on the estrous cycles.

### Default situation for regular 4-day estrous cycles

**Figure 2:**
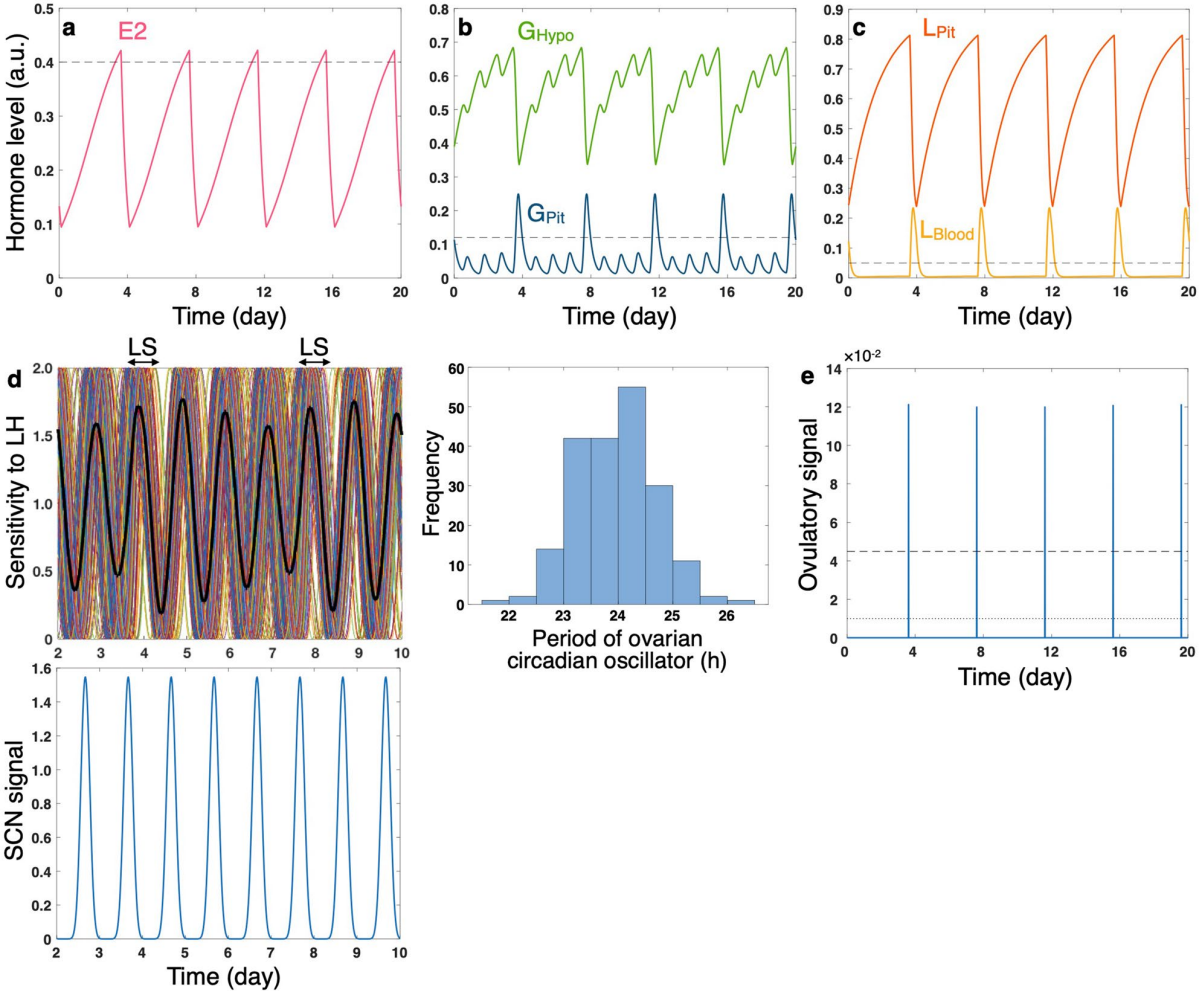
Profiles of (**a**–**c**) hormone levels, (**d**) ovarian and SCN circadian rhythms, and (**e**) ovulation dynamics simulated with parameter values generating regular 4-day estrous cycles. (**a**) Estradiol (*E2*). (**b**) GnRH in the hypothalamus (*G*_Hypo_) and pituitary (*G*_Pit_). (**c**) LH in the pituitary (*L*_Pit_) and blood (*L*_Blood_). (**d**) Ovulatory sensitivity to LH (left upper panel; color codes as in Fig. 1c) and representative data of period distribution of ovarian circadian oscillators (right panel). The SCN signal (left lower panel) is also shown for comparison of its phase with those of the ovarian oscillators. (**e**) Ovulatory signal. Black dashed lines in (**a**), (**b**), and (**c**) stand for threshold values (E^*^, G^*^, and L^*^, respectively). Dashed and dotted lines in (**e**) represent high and low values of ovulation threshold P^*^, respectively. Each time series represents a snapshot extracted arbitrarily from long-term simulation data. The simulation time does not indicate age of the animal (see also Simulation conditions in Supplementary Methods for interpretation of time).

The developed model, with adequately chosen parameter values, generates regular 4-day intervals of LS (Fig. 2). When estradiol exceeds the threshold E^*^ (Fig. 2a), large amount of GnRH in the hypothalamus is released into the pituitary, and GnRH surge occurs (hereafter referred to as GS and defined as an event in which GnRH in the pituitary exceeds the threshold G^*^; Fig. 2b). While LH in the blood basically remains in low levels, it sharply increases at the timing of GS (Fig. 2c). Estradiol decreases during the high level of LH and starts again to increase several hours after the last LS (Fig. 2a). Thanks to the entrainment of the ovarian circadian oscillators to the LH, the whole ovary’s sensitivity to LH shows high amplitude oscillation, the peak timings of which are generally phase-delayed from those of the SCN circadian signal (Fig. 2d). Ovulatory signal regularly exceeds the threshold, and estrous cycle period of 4 days is solely observed for this parameter setting (Fig. 2e).

### Decline of LH surge and resultant desynchrony of ovarian circadian oscillators are the cause of abnormal estrous cycles

Once the default situation is established, we may change values of the following four parameters and examine the estrous dynamics: *A*, amplitude of the SCN signal; ${R}_{\mathrm{G}}^{\mathrm{Act}}$, activation effect of estradiol on GnRH release; ${R}_{\mathrm{L}}^{\mathrm{Act}}$, GnRH-dependent activation effect on LH release; and *a*_E_, synthesis rate of estradiol.

**Figure 3:**
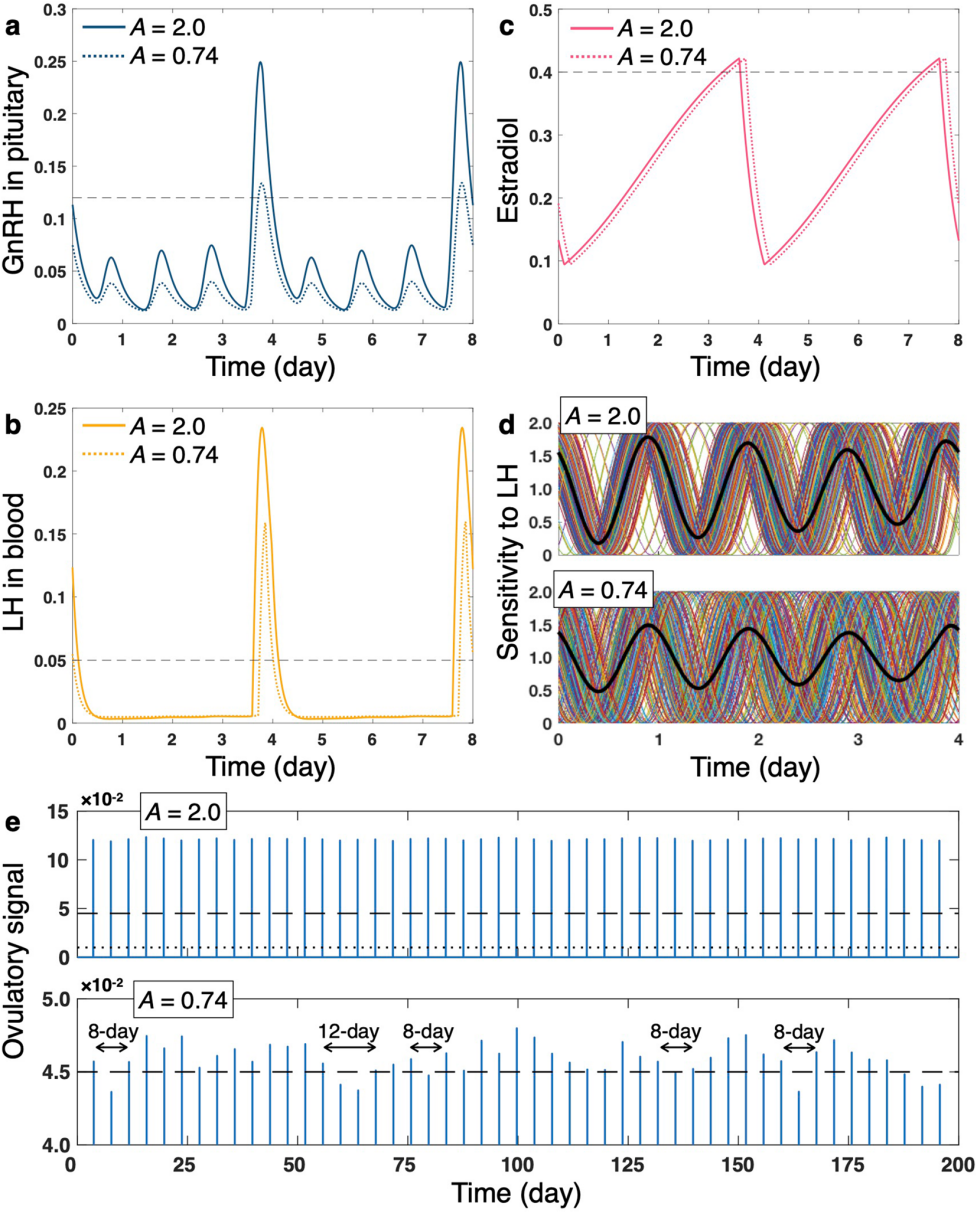
Comparison of hormonal dynamics and ovulatory property for large (*A* = 2.0, default value) and small (*A* = 0.74) amplitudes of SCN signal. (**a**) GnRH in pituitary. (**b**) LH in blood. (**c**) Estradiol. (**d**) Rhythmic ovarian sensitivity to LH in a whole ovary (black thick line) and in individual ovarian cells (other colored solid lines). (**e**) Ovulatory signal. Black dashed lines in (**a**), (**b**), and (**c**) stand for threshold values (G^*^, L^*^, and E^*^, respectively). In (**e**), dashed lines (upper and lower panels) and a dotted line (upper panel) stand for high and low ovulation threshold P^*^, respectively. Time series in (**a**–**d**) represent snapshots extracted arbitrarily from long-term simulation data. The simulation time does not indicate age of the animal (see also Simulation conditions in Supplementary Methods for interpretation of time).

When amplitude *A* of the SCN signal is set to small values (Fig. 3), GnRH release rate from the hypothalamus is reduced and, consequently, the timing at which GnRH in the pituitary exceeds the threshold G^*^ is delayed (Fig. 3a). Due to the delayed onset and reduced level of GS, LS also starts 2–3 h later than under the large *A* condition and its magnitude decreases (Fig. 3b). Although overall profiles are almost the same, phase of the estradiol dynamics is delayed compared to those with large *A* (Fig. 3c). The reduced level of LH in the blood lowers degree of synchronization between the ovarian circadian oscillators (Fig. 3d; see Supplementary Methods and Supplementary Fig. S1 for detailed analysis), causing a decline of phasic sensitivity to LH in the ovarian system as a whole. In contrast to the strong ovulatory signal observed for large *A* (Fig. 3e, upper panel), the signal is weak and variable for small *A* (Fig. 3e, lower panel). Since the ovulatory signal does not always exceed high threshold P^*^, the estrous cycles become irregular and lengthened (Fig. 3e, lower panel). Because the peak levels of LH in the small *A* condition are almost the same for different LS events (mean ± SD, 0.159 ± 6.81 * 10^–4^), the fluctuating ovulatory signal mainly stem from the desynchronization of ovarian circadian oscillators, which induces irregularity of the ovarian sensitivity to LH (even for small *A*, the ovulatory signal always exceeds small threshold P^*^).

As *A* is further lowered, LS intermittently displays 5-day intervals (Supplementary Fig. S2a). This is because sharp increase of GnRH in the pituitary does not always reach the threshold level (black arrows in Supplementary Fig. S2b) and, consequently, LS is not induced at that timing and occurs one day later (Supplementary Fig. S2a). For very small parameter values, cyclic changes in estradiol are lost, LH levels in the blood remain very low, and LS stops (Supplementary Fig. S2c).

The decrease of ${R}_{\mathrm{G}}^{\mathrm{Act}}$ or ${R}_{\mathrm{L}}^{\mathrm{Act}}$ also lowers LH levels at LS (Supplementary Fig. S3a, b) and makes the ovulatory signal variable (Supplementary Fig. S3c), although the decrease of ${R}_{\mathrm{L}}^{\mathrm{Act}}$ does not induce delayed onset of LS (Supplementary Fig. S3b). Using large values for any of *A*, ${R}_{\mathrm{G}}^{\mathrm{Act}}$, or ${R}_{\mathrm{L}}^{\mathrm{Act}}$ does not affect the rhythmicity, except for very large values of *A*, which result in abnormal daily occurrences of LS and ovulation (Supplementary Fig. S4).

The change in *a*_E_ alters intervals between estradiol surges (defined as an event in which estradiol exceeds the threshold E^*^) and, accordingly, primary intervals of the LS vary (Supplementary Fig. S5a–f). Increasing *a*_E_, in particular, shortens the intervals (*a*_E_ = 1.3 in Fig. S5a–c), which are not observed for *A*, ${R}_{\mathrm{G}}^{\mathrm{Act}}$ and ${R}_{\mathrm{L}}^{\mathrm{Act}}$. If estradiol levels are assumed to be constantly high so that they always exceed the threshold E^*^, GS and LS occur every day (Supplementary Fig. S5g–i).

### Statistical features of parameter-dependent estrous cycles

**Figure 4:**
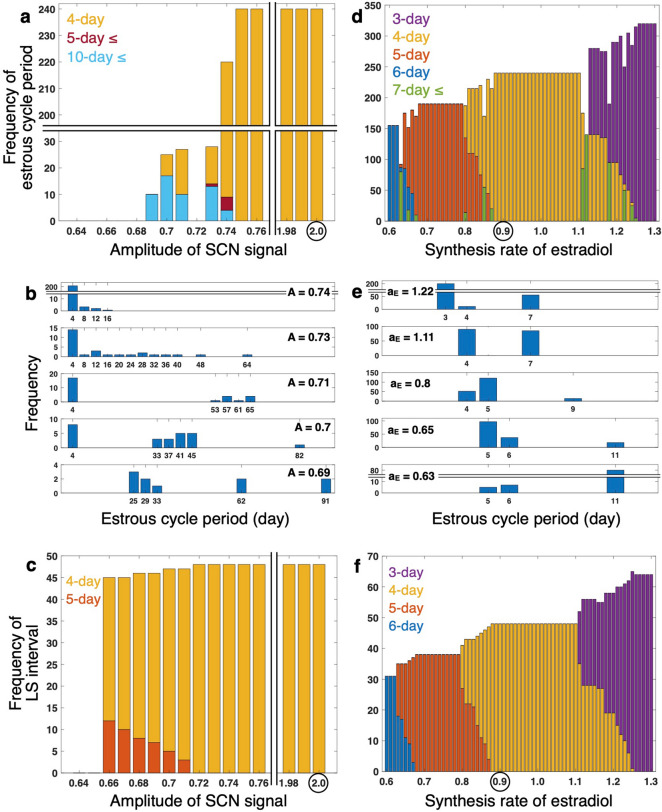
Estrous cycle period and LS interval depend on (**a**–**c**) amplitude *A* of SCN signal and (**d**–**f**) synthesis rate *a*_E_ of estradiol. (**a**, **d**) Frequency of estrous cycle periods for different parameter values. (**b**, **e**) Distribution of estrous cycle periods for representative parameter values. (**c**, **f**) Frequency of LS intervals for different parameter values. Ovulation threshold P^*^ is set to 4.5 * 10^–2^. Data are collected from five trials with different realizations of randomly-chosen ovarian circadian periods to depict (**a**), (**b**), (**d**), and (**e**). Circles in (**a**), (**c**), (**d**), and (**f**) indicate default parameter values. In (**a**) and (**c**), the estrous cycle period and the LS interval, respectively, are always 4 days between parameter values of 0.76 and 1.98.

Next, statistical features of the estrus cycles are analyzed by varying the four parameters (*A*, ${R}_{\mathrm{G}}^{\mathrm{Act}}$, ${R}_{\mathrm{L}}^{\mathrm{Act}}$, *a*_E_). The simulations are repeated for five times using different realizations of randomly-chosen periods of the ovarian circadian clocks, and the simulation data of estrous cycle periods and LS intervals are collected (Fig. 4). As detailed in the previous subsection, if smaller amplitude *A* of the SCN signal is used, irregular estrous cycles appear (especially when threshold P^*^ is high) (Fig. 4a). When *A* is set to values below 0.75, 4-day estrous cycle periods are mixed with non-4-day periods (Fig. 4a). As *A* is further decreased, cycle lengths longer than 10 days become dominant, and finally ovulation stops (Fig. 4a, b). In the LS dynamics, 4- and 5-day intervals are mixed for small *A* (Fig. 4c). Similar effects on the regularity are observed for small values of ${R}_{\mathrm{G}}^{\mathrm{Act}}$ and ${R}_{\mathrm{L}}^{\mathrm{Act}}$ (Supplementary Fig. S6). Namely, for large values of these parameters (${R}_{\mathrm{G}}^{\mathrm{Act}}$ and ${R}_{\mathrm{L}}^{\mathrm{Act}}$), ovulation and LS occur only at 4-day intervals. As the values are reduced, they occur with lengthened and multiple periods and finally stop. In the case of low threshold P^*^, estrous cycle periods are always equal to LS intervals for all three parameters (Supplementary Fig. S7a–c).

As the value of *a*_E_ is changed, the estrous cycle displays periods corresponding to the primary LS intervals or the sum of these when two distinct surge intervals are mixed (Fig. 4d, e for high threshold P^*^; Supplementary Fig. S7d for low threshold P^*^; Fig. 4f).

### Repeated change of SCN signal timing disrupts normal estrous cyclicity

When ${\psi }_{\mathrm{C}}$, the phase of the SCN signal, is repeatedly advanced or delayed for 3 h on consecutive 2 days of the week, the multi-periodicity of LS (Supplementary Fig. S8a, b) and estrous cycles (Supplementary Fig. S8c, d for high threshold P^*^; Supplementary Fig. S8e, f for low threshold P^*^) occur at larger values of *A* than animals with unperturbed ${\psi }_{C}$ (Supplementary Table S2; Fig. 4a,c; and Supplementary Fig. S7a).

### Abnormal estrous cycles caused by mutations of the circadian clock period

**Figure 5:**
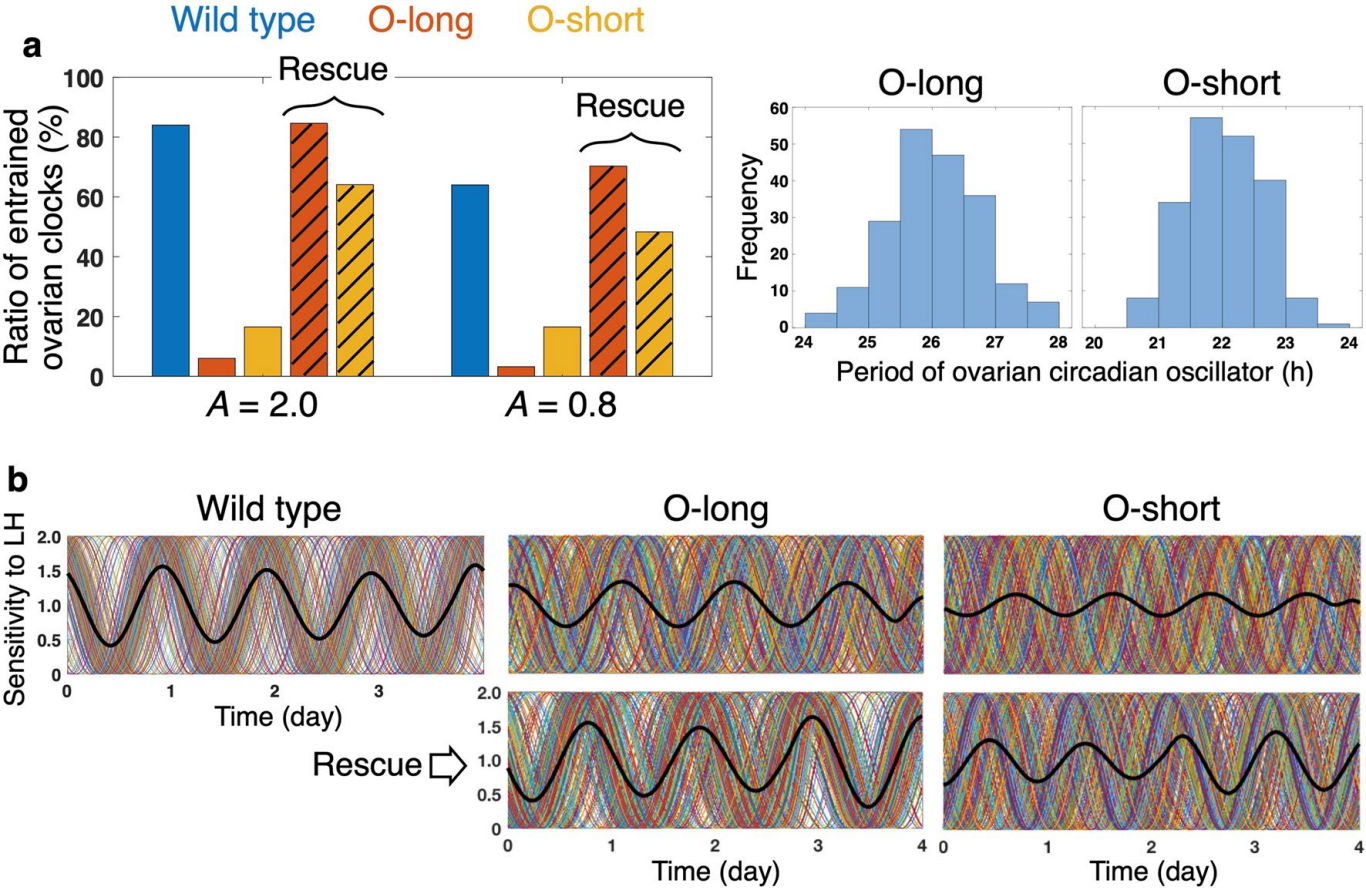
Entrainment and synchronization of ovarian circadian oscillators. (**a**) Fraction of ovarian circadian clocks entrained to LS. The data for the SCN signal with large (*A* = 2.0) and small (*A* = 0.8) amplitude are expressed as percentage in 1000 ovarian clocks (200 clocks/trial × 5 trials). Open bars indicate values obtained under 24-h environmental cycles, and hatched bars indicate values obtained from the O-long (O-short, respectively) mutant under 26-h (22-h) environmental cycles. See Supplementary Methods for evaluation of entrainment. Representative distributions of ovarian circadian periods in O-long and O-short mutants are also shown. (**b**) Rhythmic sensitivity to LH of whole ovary (black thick line) and that of individual ovarian cells (other colored solid lines). *A* is set to 0.8. In middle- and right-lower panels, the period of the SCN signal is tuned to that of the ovarian circadian oscillator. Time series in (**b**) are snapshots extracted arbitrarily from the long-term simulation data. The simulation time does not indicate age of the animal (see also Simulation conditions in Supplementary Methods for interpretation of time).

**Figure 6:**
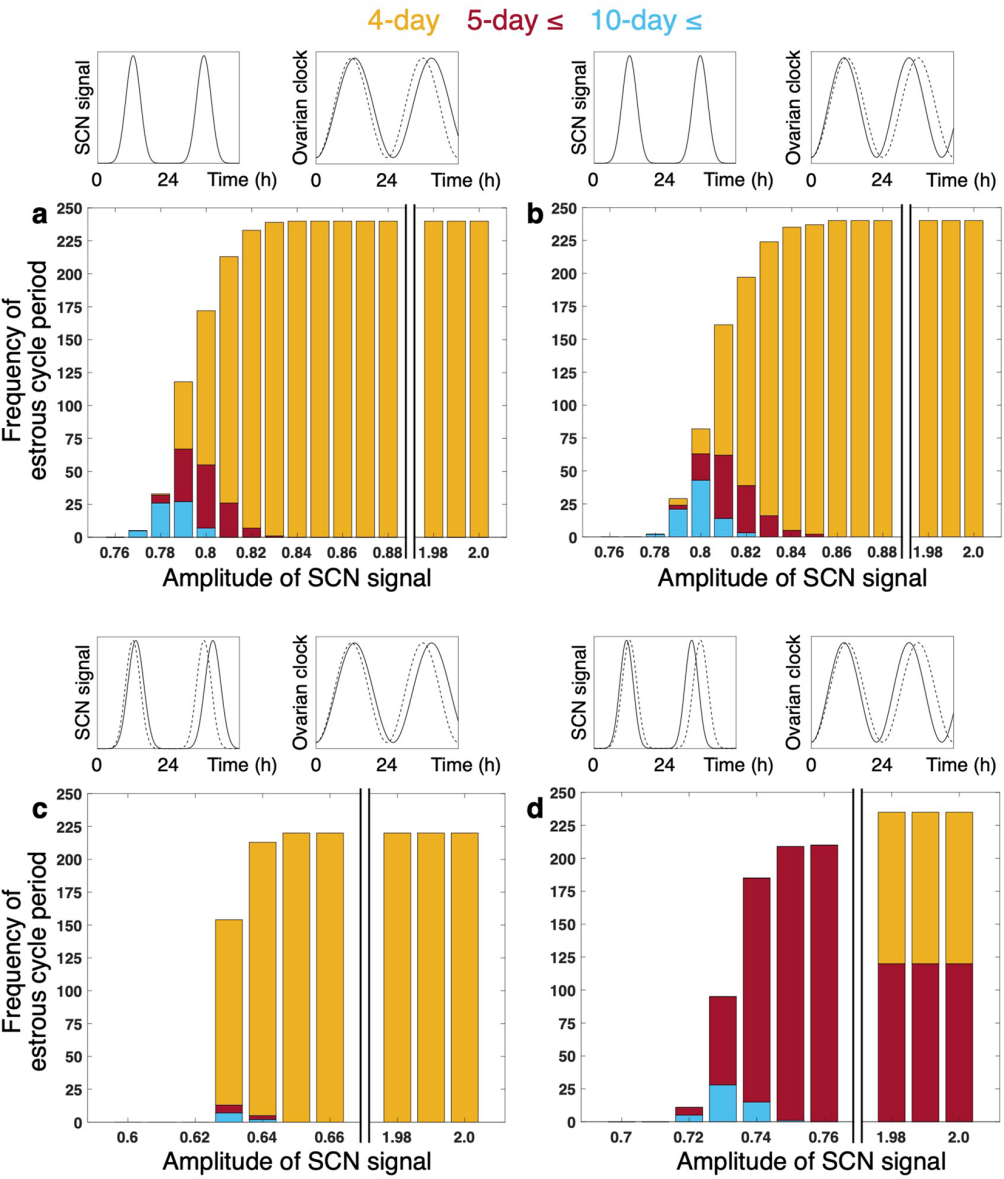
Histograms of estrous cycle periods for clock mutants under various amplitudes of SCN input. In (**a)**, (**b**), O-long and O-short mutants, respectively, receive SCN input with 24 h period. In (**c)**, (**d**), O-long and O-short mutants receive SCN input with a period corresponding to their mean FRPs. White bars in (**a**–**c**) indicate that the estrous cycle period is always 4 days in these regions. In white bar region of (**d**), estrous cycle with lengthened period or mixture of multiple periods is observed. Ovulation threshold P^*^ is set to 4.5 * 10^–2^. Data are collected from five trials with different realizations of randomly-chosen ovarian circadian periods to depict the graphs.

We next examine the impact of the circadian clock mutation on the estrous cyclicity. Particularly, the O-long and O-short mutants, in which a free-running period (FRP) of the ovarian clocks are longer and shorter, respectively, are investigated (see Simulation conditions in Supplementary Methods for details about these mutants). In these mutants, the LH stimulus does not well entrain the ovarian clocks (Fig. 5a) due to the period mismatch between these clocks and the SCN signal (see Supplementary Discussion). As a result, synchronization among the ovarian clocks is impaired and amplitude of the phasic sensitivity to LH in the ovarian system as a whole becomes small (Fig. 5a, upper panels). If high threshold P^*^ is assumed for these mutants (Fig. 6a,b), critical values of the SCN signal amplitude *A*, below which multi-period cycles (*A* = 0.84 in O-long and *A* = 0.86 in O-short) and no ovulation (*A* = 0.77 in O-long and *A* = 0.78 in O-short) appear, are both larger than those of the wild type (*A* = 0.75 and *A* = 0.69, respectively; Fig. 4a). If threshold P^*^ is low, however, the critical value of *A* to induce ovulation remains the same as that of the wild type (Supplementary Fig. S9; Supplementary Fig. S7a; Supplementary Table S2).

### Adjusted environmental-cycle length rescues the ovarian clock mutants

The value of *A*, below which no-ovulation is observed in the O-long and O-short mutants, can be lowered by adjusting an environmental cycle period to their FRP (Fig. 6c,d; Supplementary Table S2). Thanks to the coordination of the periods, the number of the entrained ovarian clocks is significantly increased compared with the mutants under the normal environmental cycle (Fig. 5a, “Rescue”). This results in the improved synchronization between the ovarian clocks and restores high-amplitude ovarian sensitivity to LH (Fig. 5b, lower panels).

## Discussion

It has been clearly demonstrated by several experiments that aging alters reproductive cyclicity^19–21^ and circadian rhythmicity^24–30^ and that altered circadian rhythmicity accelerates reproductive aging^31,32^. However, how reproductive cycles become irregular in aged animals and how impaired circadian system exacerbates reproductive aging have not yet been well understood. Our mathematical model describing the circadian-regulated ovulation predicts that the decrease of parameter values involved in the hormone dynamics and the SCN signaling results in irregular estrous cycles. This is caused by the decline of the neural output that originates from the SCN and is transduced to the ovary as a form of LS. Attenuation of the LS then gives rise to desynchronization of the ovarian circadian oscillators and disrupts the high amplitude oscillation of the ovarian sensitivity to LH. Because of the resultant misalignment between the LS and the peak ovarian sensitivity to LH, animals cannot ovulate regularly. Since, as explained below, the decrease of most of the parameters examined is relevant to aging of female reproductive functions, our results suggest that the decrease of the neural output is a potential mechanism for the loss of regular reproductive cycles in aged animals. Moreover, our model also predicts that mutation of ovarian circadian clocks further reduces their synchrony, which causes accelerated aging as detailed below. As an experiment to test our prediction, levels of synchrony among ovarian circadian oscillators and the ovulatory rhythmic sensitivity to exogenous LH should be compared between young and aged animals.

### Irregular cycles caused by the change in parameter values

Lowering a value of any of the following three parameters makes reproductive rhythms irregular and finally halts them: The SCN signal amplitude *A* (Fig. 4a–c, Supplementary Fig. S7a), estradiol-dependent activation of GnRH release ${R}_{\mathrm{G}}^{\mathrm{Act}}$ (Supplementary Fig. S6a, b, Supplementary Fig. S7b), and GnRH-dependent activation of LH release ${R}_{\mathrm{L}}^{\mathrm{Act}}$ (Supplementary Fig. S6c, d, Supplementary Fig. S7c). The decrease of these parameter values is indeed related to aging as explained in the next subsection and, therefore, we can consider animals that have smaller values of these parameters as older ones. The difference among these parameters exists in their effect on LS dynamics. Whereas the decrease in *A* and ${R}_{\mathrm{G}}^{\mathrm{Act}}$ not only attenuates LS but also delays its onset (Fig. 3b, Supplementary Fig. S3a), the timing is not affected by the reduced ${R}_{\mathrm{L}}^{\mathrm{Act}}$ (Supplementary Fig. S3b). Given the experimental fact that LH surges of aged females are characterized by both their delayed onset and reduced level^22,23^, our results suggest that physiological changes within the hypothalamus is mainly responsible for the age-related decline of LH surges. Moreover, our model also predicts that the decrease of *A* results in the phase delay of estradiol dynamics (Fig. 3c). The delayed estradiol dynamics have been indeed observed in 10- to 12.5-month-old female mice with prolonged estrous cycles^38^. Further lowering of the parameter to the value, at which LS stops, results in constant estradiol levels (Supplementary Fig. S2c). This is consistent with the experimental observation of loss of cyclic changes in serum estradiol in old, ovulation-halted rats (20–30 months old^39^).

Although no significant effect is observed for increase in the above three parameters except for very large values of *A* (Supplementary Fig. S4), increase in *a*_E_, estradiol synthesis rate, shortens periods of the reproductive rhythms (Fig. 4d–f, Supplementary Fig. S7d). Estrous cycles with a period of three days have been reported in rats^40,41^. Our model also predicts that, when estradiol levels are kept high without changing kinetic parameters of other hormones, LS occurs every day (Supplementary Fig. S5g–i), which is consistent with observations of LH surge for several consecutive days in hamsters^42^ and in rats^43^ chronically treated with high levels of estradiol. Whereas the change of *a*_E_ yields wider range of periodicities especially in LS intervals, we have not observed an arrest of LS and ovulation (Fig. 4d, f; Supplementary Fig. S7d). This suggests that the estradiol synthesis rate *a*_E_ is only slightly related to aging.

### Relationship between decreased parameter values and aging

Here we discuss how decreases in three parameters (*A*, ${R}_{\mathrm{G}}^{\mathrm{Act}}$, ${R}_{\mathrm{L}}^{\mathrm{Act}}$) can be regarded as a sign of aging. Ovariectomized middle-aged rats, when treated with estradiol, show reduction of the mRNA levels of *Kiss1* (that encodes kisspeptin) and also reduced number of kisspeptin immunopositive neurons^44,45^ compared to young rats treated in the same manner. In our model, such decreased release of the GnRH secretagogue kisspeptin can be represented by decrease in estradiol positive-feedback effect on GnRH release, parameterized by ${R}_{\mathrm{G}}^{\mathrm{Act}}$.

Experimental observations both in vivo^46,47^ and in vitro^48,49^, in which pituitaries of aged rats release less LH in response to exogenous GnRH, support the notion of decreased ${R}_{\mathrm{L}}^{\mathrm{Act}}$, the GnRH-dependent activation effect on LH release.

Regarding the amplitude *A* of the SCN signal *C*, we consider the signal *C* as a net input to GnRH neurons, which depends on both the gated response of the neurons to signal peptides such as kisspeptin and daily variations in the peptide itself^8^. The decrease of *A* and the resultant reduced magnitude of *C* (Fig. 1b) can be, therefore, caused by several ways. For example, desynchronization between signal-peptide secreting neurons and GnRH neurons can cause mismatch between secretion of high-level peptides and the high sensitivity of GnRH neurons. Age-related decline of circadian outputs from the SCN such as the neurotransmitter VIP^30^ and neural activity of the SCN itself^24^ may potentially disrupt synchronization between hypothalamic neurons as revealed for neurons within the SCN^24^. Another possibility is decrease of the abundance of signal peptides or of the responsiveness of GnRH neurons to them, which clearly leads to the weakened SCN signal. This possibility, however, is not very likely since, in aged rats, (i) the circadian expression of AVP, the important neurotransmitter affecting on kisspeptin neurons, is not altered ^30^, implying unaltered kisspeptin release, and (ii) application of exogenous kisspeptin increases the LH surge release to levels comparable to those of young rats^44^, implying unaltered sensitivity of the GnRH neurons to kisspeptin.

### Effects of the timing of the SCN signal on the estrous cyclicity

Not only the amplitude, but also the timing of the SCN signal is crucial for the realization of regular estrous cycles. If ${\psi }_{\mathrm{C}}$, the phase of the SCN signal, is weekly changed by 3 h over 2 days, estrous cycles display multi-periodicity at larger values of *A* than under the normal condition (Supplementary Table S2). This result suggests that the perturbation that potentially alters the timing of the neural circadian signal disrupts regular estrous cyclicity, which animals could show under normal environmental conditions. Takasu et al. (2015)^32^ have indeed observed that middle-aged (8–12 months old) mice, which display regular estrous cycles under normal LD (12-h light:12-h dark) cycles, show irregular estrous cycles when they are exposed to weekly perturbed environmental conditions.

It has been suggested that the strong circadian input triggering LH surges in vivo is restricted to a duration of a few hours (“critical period”) of proestrus^50^. This implies that, for circadian regulation of the estrous cycles, the SCN signal should fall within the critical period, which is defined in our model by the default value of ${\psi }_{\mathrm{C}}$. The simulated irregular estrous cycles induced by the weekly perturbation of ${\psi }_{\mathrm{C}}$ can be interpreted as misalignment between the timing of the SCN signal and the estrous cycles.

### Abnormal estrous cycles of ovarian-clock mutants and their rescue by the adjustment of environmental cycles

Under 24-h environmental cycles, the O-long and O-short mutants, in which a mean FRP of the ovarian clock is long (26 h) and short (22 h), halt their ovulation at a larger value of *A* than the wild type (Supplementary Table S2; see also Supplementary Discussion). This result indicates that, under such environmental condition, aging is accelerated in the O-long and O-short mutants since larger values of *A* correspond to younger animals as explained above. Pilorz and Steinlechner (2008)^31^ have reported emergence of the irregular estrous cycles at an earlier age than wild-type mice in *Per1* and *Per2* mutants, both of which display short FRP phenotypes^51,52^. The accelerated aging of reproduction has also been observed in short-FRP *Cry1* and long-FRP *Cry2* mutants^32^. Takasu et al. (2015)^32^ have further reported a restoration of regular estrous cycles in the *Cry* mutants by tuning the length of the environmental LD cycle to their FRP. Such prevention of the early appearance of the estrous abnormality by the adjustment of environmental conditions can be reproduced by our model: The critical value of *A*, below which no-ovulation is observed, becomes low when the environmental cycle period is equated to the FRP of the O-long or O-short mutant (Supplementary Table S2). These phenomena can be explained by the improved entrainment of the ovarian clocks to the adjusted environmental cycles (Fig. 5, “Rescue”). On the other hand, even when *A* is large, i.e. animals are young, the O-short mutants under the short environmental cycle display estrous cycles in which 4- and 5-day periods are mixed (Fig. 6d). This result is inconsistent with the experimental observation of the *Cry1* mutants that stably exhibit estrous cycles with a single period (4 or 5 days) under the short LD cycle^32^. To resolve this discrepancy, we make an additional assumption that the rate of hormone production is adjusted in response to the change of the LD-cycle length (Supplementary Methods). Under this condition, the O-short mutants under the short LD cycle show regular 4-day estrous cycles when *A* is large (Supplementary Fig. S10).

### Different ovulatory tendency leads to distinct estrous cyclicity

Throughout almost all simulations, we have compared the results between large and small values of P^*^ (ovulation threshold). Generally speaking, the results are more drastic for high P^*^. For instance, only when P^*^ is high, small amplitude of the SCN signal causes estrous cycles longer than 10 days in the wild type (Fig. 4a for high P^*^; Supplementary Fig. S7a for low P^*^) and in the ovarian-clock mutants (Fig. 6a,b for high P^*^; Supplementary Fig. S9 for low P^*^). The dysfunction of reproductive cycles has been observed experimentally in old wild-type rats and mice^19–21^ and in circadian-clock mutants^31,32^. These observations, together with our simulation results, imply a relatively high ovulation threshold in rodents. Such a high threshold enables ovulation to occur only when the ovulation-inducing signal, potentially determined by LH surge levels and the ovarian sensitivity to it, is significantly large. In our simulation of the wild type with high P^*^, extremely long estrous-cycle period of about 90 days has been observed (Fig. 4b). This is in good contrast with the experimental studies that report cycle length of up to 40 days in rats^40,41^. Our model prediction of the very long period is partly due to a high value set to P^*^, which affects the cycle length as mentioned above, and does not necessarily mean that our model misses crucial components for regulating the estrous cyclicity.

## Methods

Here we explain our mathematical modelling of the circadian-system-regulated estrous cycles. Briefly, the model contains five variables for the hormonal dynamics and *N* variables for a population of ovarian circadian oscillators (*N*: number of ovarian cells). Hormone synthesis and clearance are basically described by linear equations except for the estradiol synthesis, which is modeled by a logistic growth equation. The model also includes three thresholds, which define surge states of the estradiol, GnRH, and LH. A population of ovarian circadian oscillators is heterogeneous in terms of their FRPs, and their desynchronization due to the reduced LH input results in irregular estrous cycles as described in “Results” and “Discussion”. In Supplementary Methods, we explain detailed simulation conditions including how to simulate circadian-clock mutants and how to determine the parameter values.

### Hormone dynamics in hypothalamus, pituitary and peripheral blood

We assume that synthesis of *G*_Hypo_, GnRH in the hypothalamus, depends on its current level and decreases to zero as the level approaches a threshold. Similar assumption has been made in the previous bovine estrous model^35^. Release of GnRH from the hypothalamus basically occurs in a pulsatile manner (approximately 1 pulse/h^53^) and a change of its rate, in the presence of estradiol, shows a clear circadian rhythm in cocultures of the SCN and preoptic area containing GnRH neurons^54^. Because in the current study we investigate the event that takes place at intervals of several days (i.e. ovulation), we neglect the relatively fast dynamics of the pulsatile release, although such dynamics have been also investigated mathematically^55,56^. Funabashi et al. (2000)^54^ have also reported that, when estradiol is absent, GnRH is continuously released but the circadian rhythm of the release rate disappears, suggesting that the circadian regulation is dependent on the presence of the estradiol. We thus consider two components of the GnRH release; one is a constant component with a rate *r*_G_ and the other a SCN-derived rhythmic component. The rhythmic component is determined by both the circadian effect *C*, which is a function of time and is detailed later, and the estradiol effect *R*_G_, which is a function of the estradiol level *E2*. Estradiol exerts, mainly via kisspeptin neurons with estrogen receptors, both positive and negative effects on GnRH neurons: Their neural activity is repressed when estradiol levels are low, while it is activated when its levels are high^4^. These dual effects of estradiol are modeled by the function *R*_G_ that switches between two rate constants ${R}_{\mathrm{G}}^{\mathrm{Act}}$ and ${R}_{\mathrm{G}}^{\mathrm{Rep}}$ (${R}_{\mathrm{G}}^{\mathrm{Act}}>{R}_{\mathrm{G}}^{\mathrm{Rep}}$) depending on whether or not the estradiol level exceeds a threshold E^*^. We assume that the effects of estradiol exert on GnRH release with a time delay *τ*_E_. These processes are integrated into the following equations:

1 $$\frac{d}{dt}{G}_{\mathrm{Hypo}}\left(t\right)={a}_{\mathrm{G}}\left(1-\frac{{G}_{\mathrm{Hypo}}}{{G}_{\mathrm{Max}}}\right)-{(r}_{\mathrm{G}}+{R}_{\mathrm{G}}\left(E2\right)C(t)){G}_{\mathrm{Hypo}},$$

with

2 $${R}_{\mathrm{G}}\left(E2\right)=\left\{\begin{array}{c}{R}_{\mathrm{G}}^{\mathrm{Act}} \quad E2(t-{\tau }_{\mathrm{E}})\ge {E}^{*}\\ {R}_{\mathrm{G}}^{\mathrm{Rep}} \quad E2(t-{\tau }_{\mathrm{E}})<{E}^{*}\end{array}\right.,$$

where *a*_G_ is a GnRH synthesis rate and *G*_Max_ is a synthesis threshold. Regarding the SCN-derived circadian signal *C*, the in-vitro study mentioned above has reported a sinusoidal-like pattern of GnRH release^54^, implying that neural timing signals are also sinusoidally transduced. In addition, the potent circadian input leading to LH surges in vivo seems to be limited to a narrow time window of a day^50^, suggesting that, only during that time window, the neural signals are strong enough to induce the release of a large amount of GnRH and the resultant LH surge. Based on these experimental observations, we define *C* by a function that is based on a sinusoidal wave and is constructed to reproduce the suggested sharpness. It has been also observed that, when female hamsters are exposed to non-24-h LD cycles, their estrous cycle length is changed accordingly and, in most cases, becomes very close to quadruple multiple of a period of the LD cycles^57^. Because in the current study we focus on the ovulation dynamics under LD cycles, we assume that the SCN signal *C* oscillates with a period *T* of environmental cycles and define it as follows (Fig. 1b):

3 $$C\left(t\right)=A{\left\{{a}_{\mathrm{C}}+{b}_{\mathrm{C}}\mathrm{sin}\left({\omega }_{\mathrm{C}}t-2\pi {\nu }_{\mathrm{C}}\frac{{\psi }_{\mathrm{C}}}{24}\right)\right\}}^{n},$$

with

4 $${\omega }_{\mathrm{C}}=\frac{2\pi }{T},$$

where *A* stands for amplitude, *a*_C_ and *b*_C_ are constants satisfying *a*_C_ > *b*_C_, *ω*_C_ is a frequency, ${\psi }_{\mathrm{C}}$ controls the peak timing and is scaled to a period *T* by a factor ${\nu }_{\mathrm{C}}=T/24$, and *n* determines sharpness of *C*. *C* can be considered as a net circadian input to the GnRH neurons, namely, it integrates the effect of a signal peptide (e.g. kisspeptin) and the neuronal responsiveness to the peptide, both of which have been suggested in hamsters to exhibit daily variations^8^.

GnRH in the pituitary (*G*_Pit_) is cleared at a constant rate *c*_G_:

5 $$\frac{d}{dt}{G}_{\mathrm{Pit}}(t)={(r}_{\mathrm{G}}+{R}_{\mathrm{G}}\left(E2\right)C(t)){G}_{\mathrm{Hypo}}-{c}_{\mathrm{G}}{G}_{\mathrm{Pit}}.$$

For synthesis of *L*_Pit_, LH in the pituitary, we make the same assumption as that for *G*_Hypo_ synthesis and again use the logistic-type equation with two parameters *a*_L_ (LH synthesis rate) and *L*_Max_ (synthesis threshold). Except for late afternoon of proestrus at which the LH surge occurs, plasma LH levels are very low throughout estrous cycles^58^. This is at least partly because of negative feedback effect of estrogen on gonadotroph^59^ and the effect of gonadotropin-inhibitory hormone (GnIH) decreasing the activity of GnRH neurons^60^. Although several factors including increase of GnRH receptors in the pituitary^61^ and positive effect of estradiol on gonadotroph^59^ have been implicated in the occurrence of the LH surge, the surge release of GnRH seems to be indispensable to the LH surge^53,62^. We thus model the drastic change of the LH release dynamics by a function *R*_L_ that depends only on the GnRH level in the pituitary and switches between two rate constants ${R}_{\mathrm{L}}^{\mathrm{Act}}$ and ${R}_{\mathrm{L}}^{\mathrm{Rep}}$ (${R}_{\mathrm{L}}^{\mathrm{Act}}>{R}_{\mathrm{L}}^{\mathrm{Rep}}$). ${R}_{\mathrm{L}}^{\mathrm{Act}}$ and ${R}_{\mathrm{L}}^{\mathrm{Rep}}$ can be considered as parameters integrating the activation and repression effects, respectively, on LH release mentioned above. We also introduce constant release with a rate *r*_L_. These processes are formalized as follows:

6 $$\frac{d}{dt}{L}_{\mathrm{Pit}}(t)={a}_{\mathrm{L}}\left(1-\frac{{L}_{\mathrm{Pit}}}{{L}_{\mathrm{Max}}}\right)-{(r}_{\mathrm{L}}+{R}_{\mathrm{L}}\left({G}_{\mathrm{Pit}}\right)){L}_{\mathrm{Pit}},$$

with

7 $${R}_{\mathrm{L}}\left({G}_{\mathrm{Pit}}\right)=\left\{\begin{array}{c}{R}_{\mathrm{L}}^{\mathrm{Act}} \quad  {G}_{\mathrm{Pit}}\ge {G}^{*}\\ {R}_{\mathrm{L}}^{\mathrm{Rep}} \quad {G}_{\mathrm{Pit}}<{G}^{*}\end{array}\right.,$$

where G^*^ is a threshold. The duration, during which *G*_Pit_ is equal to G^*^ or greater, is referred to as GS to be distinguished from GnRH surges in general context.

LH in the blood (*L*_Blood_) is cleared at a rate *c*_L_:

8 $$\frac{d}{dt}{L}_{\mathrm{Blood}}(t)={(r}_{\mathrm{L}}+{R}_{\mathrm{L}}\left({G}_{\mathrm{Pit}}\right)){L}_{\mathrm{Pit}}-{c}_{\mathrm{L}}{L}_{\mathrm{Blood}}.$$

We use a term LS to specify LH surges in the model and assume that LS begins when *L*_Blood_ reaches a threshold L^*^. The onset time of the *i*th LS event is termed ${t}_{i}^{\mathrm{On}}$ (Fig. 1c).

Estradiol in rodents with 4-day estrous cycles exhibits relatively simple dynamics: It increases almost exponentially throughout each estrous cycle and sharply decreases for about half a day after LH surges^58^. Moreover, both the age^63^ and disruption of circadian clocks^64,65^ do not largely affect estradiol dynamics. They are thus modeled by two simple ordinary differential equations, a logistic growth equation for the increase phase and an exponential decay equation for the decrease phase. The decrease phase starts at ${t}_{i}^{\mathrm{On}}$ and lasts for *τ*_LS_ hours:

9 $$\frac{d}{dt}E2\left(t\right)=\left\{\begin{array}{*{20}l}-{c}_{\mathrm{E}}E2 & \quad {t}_{i}^{\mathrm{On}}\le t<{t}_{i}^{\mathrm{On}}+{\tau }_{\mathrm{LS}}\\ {a}_{\mathrm{E}}E2\left(1-\frac{E2}{{E}_{\mathrm{Max}}}\right) &\quad \text{otherwise}\end{array}\right.,$$

where *c*_E_ and *a*_E_ are rate constants for clearance and synthesis, respectively, and *E*_Max_ stands for a maximum estradiol level.

### Circadian oscillators in ovarian cells and ovulation

Rodents generally ovulate approximately 12 h after LH surges^3,66^. In addition, ovulation depends not only on phasic sensitivity to LH in the ovary^16,17^ but also on LH surge levels: Greig and Weisz (1973)^67^ have shown that more ova tend to be produced if LH levels at proestrus are higher. We here consider “ovulatory signal” *P* and assume that ovulation occurs if *P* reaches a threshold P^*^ (Fig. 1c). *P* is dependent both on the rhythmic ovarian sensitivity *S* to LH and on *L*_Blood_ for *τ*_P_ hours after the onset of LS. The ovulatory signal *P*_*i*_ resulting from the *i*th LS event is thus given by the following integral:

10 $${P}_{i}={\int }_{{t}_{i}^{\mathrm{On}}}^{{t}_{i}^{\mathrm{On}}+{\tau }_{\mathrm{P}}}{L}_{\mathrm{Blood}}(t)S(t)dt.$$

The value of P^*^ determines the tendency of animals to ovulate: If P^*^ is set to smaller values, animals could ovulate with *P*_*i*_ for which animals having larger P^*^ cannot ovulate. We speculate that such an intrinsic tendency would contribute to variations in the regularity of estrous cycles within the same age group^21^. In the current study we utilize two different values of P^*^ (Fig. 1c; Supplementary Table S1) and examine how it affects the estrous cyclicity. Because the diurnal rhythm of ovulatory response to LH displays a sinusoidal-like pattern^16,17^, we model *j*th ovarian cell’s rhythmic sensitivity to LH, denoted as *S*_*j*_, by a sinusoidal function of its circadian phase *φ*_*j*_:

11 $${S}_{j}\left({\varphi }_{j}\right)={a}_{\mathrm{S}}+{b}_{\mathrm{S}}\mathrm{sin}\left({\varphi }_{j}-2\pi {\nu }_{\mathrm{S}}\frac{{\psi }_{\mathrm{S}}}{24}\right),$$

where *a*_S_ and *b*_S_ are constants satisfying *a*_S_ ≥ *b*_S_, and ${\psi }_{\mathrm{S}}$ determines the peak of *S*_*j*_ and is scaled to an oscillation period *τ*_*j*_ of the *j*th cell by a factor ${\nu }_{\mathrm{S}}={\tau }_{j}/24$. The rhythmic sensitivity *S* of the ovary as a whole is given by an average of *S*_*j*_ over all ovarian cells, i.e. $S=(1/N){\sum }_{j=1}^{N}{S}_{j}$, where *N* stands for the number of the cells. Modeling the circadian phase is based on a couple of experimental observations by Yoshikawa et al. (2009)^18^. First, LH shifts the phase of a *Per1-luc* expression rhythm of ovarian granulosa cells in a time-dependent manner. Second, individual granulosa cells display different peak phases of the rhythm, suggesting that ovarian circadian oscillators are not well synchronized with each other probably due to weak, if any, coupling between the cells. The phase *φ*_*j*_ of the *j*th circadian oscillator is thus defined by the following uncoupled phase oscillator model:

12 $$\frac{d}{dt}{\varphi }_{j}\left(t\right)={\omega }_{j}+\gamma {Z}_{j}\left({\varphi }_{j}\right){L}_{\mathrm{Blood}}$$

with

13 $${\omega }_{j}=\frac{2\pi }{{\tau }_{j}}$$

and

14 $${Z}_{j}\left({\varphi }_{j}\right)=\mathrm{sin}\left({\varphi }_{j}-2\pi {\nu }_{\mathrm{Z}}\frac{{\psi }_{\mathrm{Z}}}{24}\right),$$

where *ω*_*j*_ and *Z*_*j*_ are, respectively, natural frequency and phase sensitivity function (or infinitesimal PRC) to the LH stimulus of the *j*th circadian oscillator, ${\psi }_{\mathrm{Z}}$ determines the peak of *Z*_*j*_ and is scaled to an oscillation period *τ*_*j*_ of the *j*th oscillator by a factor ${\nu }_{\mathrm{Z}}={\tau }_{j}/24$, and a constant *γ* controls strength of the LH effect on the phase. The oscillation periods *τ*_*j*_ are normally distributed with mean *τ*_O_ and standard deviation of 3% of the mean. Since magnitude of phase shifts induced by exogenous LH depends on its dose^18^, we simply use values of *L*_Blood_ for the LH effect without introducing any nonlinear modulation, which can be modeled by e.g. the Hill function.

## Supplementary information

Supplementary Information.

## Abbreviations

GS: GnRH surge in the model
LS: LH surge in the model
