# Supplementary material for: Modeling circadian regulation of ovulation timing: age-related disruption of estrous cyclicity
Source: Sci Rep. 2020 Oct 7;10:16767. doi: 10.1038/s41598-020-73669-x (PMC7541497; doi:10.1038/s41598-020-73669-x)
Supplement: Supplementary file 1 — Supplementary Information. [file 41598_2020_73669_MOESM1_ESM.pdf]

Supplementary Information

Modeling circadian regulation of ovulation timing: Age-related disruption of estrous cyclicity

Takayuki Ohara[1,*], Takahiro J Nakamura[2], Wataru Nakamura[3], Isao T Tokuda[4,*]

[1]Institute of Genetics and Biometry, Leibniz Institute for Farm Animal Biology

[2]Laboratory of Animal Physiology, School of Agriculture, Meiji University

[3]Department of Oral-Chrono Physiology, Graduate School of Biomedical Sciences, Nagasaki University

[4]Department of Mechanical Engineering, Ritsumeikan University

[*]Correspondence and requests for materials should be addressed to T.O. (email: ohara@fbn-dummerstorf.de) or to I.T.T. (email: isao@fc.ritsumei.ac.jp)

Supplementary Methods

Simulation conditions

In the following we explain simulation conditions including how to simulate aging or circadian-clock-mutants and how to determine parameter values.

Simulation of aging and clock mutants

Among various hormones considered in the developed model, LH dynamics on proestrus have been well studied in terms of the age-related alteration: In aged rats LH surges start 1–3 hours later and show the lower level [1, 2], which could affect the estrous cyclicity. We thus evaluate the impact of the following four parameters, which can directly or indirectly affect the LH dynamics, on the estrous cyclicity: A, the amplitude of the SCN-derived circadian signal, a_{E}, estradiol synthesis rate, $R_{\mathrm{G}}^{\mathrm{Act}}$, activation effect of estradiol on GnRH release, and $R_{\mathrm{L}}^{\mathrm{Act}}$, GnRH-dependent activation effect on LH release. By using smaller or larger parameter values, we observe how the ovulation dynamics are altered.

We also examine the estrous cyclicity of the circadian clock mutants. The mutants having circadian clocks with a short or long free-running period (FRP) such as *Cry1* and *Cry2* mutants display irregular estrous cycles at an earlier age than wild-type mice [3]. The observed combined effect of aging and circadian deficiency on estrous cyclicity is of great interest and is adequate to be studied here: We thus simulate these mutants. It is very likely under light-dark (LD) cycles that, even when the FRP of the SCN clocks is shortened or lengthened, the clocks oscillate with the same period as that of the environmental cycle because of the strong zeitgeber effect of light. Therefore, we investigate the situation where the FRP of only the ovarian clock is shortened or lengthened, which we call O-short or O-long mutants, respectively. They are simulated by using a small or a large value of τ_{O}, a mean period of a population of ovarian circadian oscillators.

Simulation of social jetlag and rescue experiments

We further address the relationship between the circadian system and estrous cyclicity in the context of the environmental change. To this end, we simulate two kinds of experiments performed by Takasu et al. (2015) [3]. The first experiment is involved with the so-called social jetlag [4, 5], in which animals are weekly perturbed by a 3-hour shift of onset of darkness for consecutive 2 days. To simulate this experiment, we switch between the 5-day duration accompanied with a default value of the SCN signal phase ψ_{C} (see below for the determination of the default value) and the 2-day duration with a 3-hour advanced or delayed ψ_{C}. In the second experiment, estrous cycles of short and long FRP mutant mice (*Cry1*$^{-/-}$ and *Cry2*$^{-/-}$, respectively) have been examined under the LD cycle with the cycle length adjusted to the FRP of each mutant [3]. We examine the ovulation dynamics of the O-long and O-short mutants by setting

the environmental-cycle period T to the same value as τ_O of each mutant so that the SCN and ovarian clocks oscillate with the same period.

For each mutant, we again examine the impact of parameter values on estrous cyclicity. As shown in Results of the main text, smaller values of the above-mentioned parameters induce basically a similar variation in the estrous cyclicity of the wild type. We therefore focus only on the influence of smaller parameter values and choose A as the representative.

In Results of the main text, the estrous cycle period is expressed as the number of days, where the length of one day corresponds to that of the environmental cycle (i.e. T). It turns out that, even in the wild type, the estrous cycle period is not always an integer multiple of T and, for specific parameter values, it displays a small deviation. We thus tolerate deviation of a half of T from the expected integer multiple of T.

Default parameter setting

Values of the three parameters that control phases of the SCN signal (ψ_C; equation (3) in the main text), rhythmic sensitivity to LH (ψ_S; equation (11) in the main text) and phase sensitivity function (ψ_Z; equation (14) in the main text) are determined by seeking for their combination that maximizes the ovulatory signal P (equation (10) in the main text) of a single ovarian cell (i.e. the number of the ovarian cells N is set to 1). For this optimization, T and τ_O (length of the environmental cycle and a mean period of the ovarian circadian oscillators, respectively) are set to 24 hours. Each phase parameter is changed from 0 to 22 hours in increments of 2 hours. In total, 12^3 parameter combinations are sought.

In our simulation study, T is set to 24 hours unless otherwise stated. τ_O is set to 26 and 22 hours for the O-long and O-short mutants, respectively, and to 24 hours for the wild type. Default values of other parameters are chosen so that the model reproduces regular 4-day estrous cycles in young wild-type rodents (Supplementary Table S1). Although we report the results with the initial condition, under which $E2$, G_{Pit}, and L_{Blood} are not at their surge state at $t = 0$, we have confirmed that other choice of the initial condition provides qualitatively similar results. We have also confirmed that our main results are not largely affected by variations of parameters including a_G and a_L, synthesis rates of GnRH in hypothalamus and LH in pituitary, respectively, and c_G and c_L, clearance rates of GnRH in pituitary and LH in blood, respectively.

Transient process

The simulation is performed for 500 days, where data for the first 300 days are discarded to avoid transients and data for the remaining 200 days are used for the analyses. Each simulation is repeated for five times with different realization of the randomly chosen ovarian clock periods. It should be noted that, in our simulation, the aging effect is simulated only by the usage of different values of A, a_E, $R_{\mathrm{G}}^{\mathrm{Act}}$, and $R_{\mathrm{L}}^{\mathrm{Act}}$, which are set at the beginning of each

simulation. The animal at day 0 and day 500 in a given simulation has exactly the same values of these physiological parameters. The former animal, therefore, cannot be interpreted as a newborn and the latter cannot be interpreted as an aged animal. Each simulation result is rather interpreted as a long-term observation of the estrous dynamics of an animal that is assumed not to age in terms of the lapse of time. The relationship between the aging and the change of these parameters is explained in Discussion in the main text. The delay differential equations are solved by an algorithm based on the solver *dde23* of MATLAB (R2018a; MathWorks), which is slightly modified to monitor the timing of LS.

Quantification of synchrony between ovarian circadian oscillators

To quantify degree of synchronization between ovarian circadian oscillators, synchronization index $SI(t)$, which is known as the Kuramoto order parameter [6], is calculated as follows:

$$SI(t) = \frac{1}{N}\left|\sum_{j=1}^{N} \exp\,(i\varphi_j)\right|, \tag{S1}$$

where N stands for the number of the oscillators and φ_j is a phase of the jth oscillator. $SI = 1$ implies complete synchrony, while $SI = 0$ indicates desynchrony.

In the developed model, the LH stimulus to the phase of the ovarian clock is very strong at LS and is very weak otherwise, because its strength is proportional to the LH level in the blood (equation (12) in the main text). SI is accordingly large at LS events and gradually decreases between them, generating oscillation patterns (Supplementary Fig. S1). SI calculated with large amplitude of the SCN signal is consistently larger than that with small amplitude (Supplementary Fig. S1).

Detection of entrainment of ovarian circadian oscillators to LH stimulus

To examine whether individual ovarian circadian oscillators is entrained to strong LH stimulus at LS, we investigate how coherent the circadian phase is at LS events for each oscillator. If an ovarian clock is stably entrained, its phase is expected to take on similar values for consecutive LS events. We define an entrainment index EI_j of the jth circadian oscillator as

$$EI_j = \frac{1}{n}\left|\sum_{k=1}^{n} \exp\left(i\varphi_j(t_k^{On})\right)\right|, \tag{S2}$$

where n stands for the total number of LS events in one simulation, φ_j is the phase of the jth oscillator, and t_k^{On} is the onset time of the kth LS event. EI_j is equivalent to the Kuramoto order parameter for the LS onset phase. $EI_j = 1$ implies perfect entrainment, while $EI_j = 0$ indicates no coherence of the phase. In Fig. 5a, the jth ovarian clock is considered to be entrained if EI_j is equal to or greater than 0.8.

Rescue simulation of the O-short mutants with the assumption of the adjustable hormone production rate

The O-short mutants under the short environmental cycle display irregular estrous cycles even when A is large (Fig. 6d). This result is not consistent with the experimental observation of the *Cry1* mutants that exhibit the regular cycles under the short LD cycle [3]. To resolve this discrepancy, we infer that the hormonal dynamics could also be adjusted in response to the change of the LD-cycle length, although, to our knowledge, such adjustment has not been experimentally observed. Given the importance of estradiol feedback to LH surge induction, we assume that the rate of estradiol synthesis is adjusted to the LD-cycle length. In this case equation (9) for estradiol dynamics in the main text is modified as

$$\frac{d}{dt}E2(t) = \begin{cases} -c_{\mathrm{E}}E2 & t_i^{\mathrm{On}} \leq t < t_i^{\mathrm{On}} + \tau_{\mathrm{LS}} \\ \frac{24}{T}a_{\mathrm{E}}E2\left(1 - \frac{E2}{E_{\mathrm{Max}}}\right) & \text{otherwise} \end{cases}. \tag{S3}$$

The O-short mutants with this additional assumption display regular 4-day estrous cycles under the short environmental cycle when A is large (Supplementary Fig. S10). The critical value of A, below which ovulation is not observed anymore, is lower than that in the O-short mutants under the 24-h environmental cycle (Fig. 6b).

Although these simulations are intended as mutants, we can regard those as wild type animals under non-24-hour environmental cycles, where their central and peripheral circadian clocks are entrained to the environmental cycles. Carmichael et al. (1981) [7] have reported that, when young hamsters are exposed to LD cycles shorter than 24 hours, the estrous cycle period becomes very close to the quadruple of the LD cycle length. Assuming that central and peripheral circadian clocks of these hamsters are entrained to the environmental cycles, our model suggests that estradiol dynamics are under the control of the circadian system and their kinetic parameters are regulated to adjust to the environmental change.

Supplementary Discussion

The reduced capability of ovulation in the O-long and O-short mutants

We here explain why multi-period estrous cycles and an arrest of ovulation are observed at larger values of A in the O-long and O-short mutants than in the wild type. These mutants have a mean FRP of the ovarian circadian clock, either longer (26 hours) or shorter (22 hours) than that of the wild type. Since their SCN signal still oscillates with the period of 24 hours, phases of the SCN signal and ovarian clocks deviate from each other by 2 hours in every circadian cycle. This means that LS, the timing of which is determined by the phase of the SCN signal, does not always occur at the same timing in terms of the ovarian-clock phase. Since external stimuli, in general, have to act on the same (or at least similar) phase of an oscillator in order to establish stable entrainment, the LH stimulus at LS cannot well entrain the mutated individual ovarian clocks (Fig. 5a; see Supplementary Methods for detailed explanation of analysis of entrainment). The phases of the individual ovarian clocks become far apart from each other, resulting in the more flattened rhythmic sensitivity of the mutant to LH than that of the wild type (Fig. 5b). These harmful effects on the induction of ovulation become evident as the direct contribution of the LH level to the ovulatory signal (see equation (10) in the main text) is decreased by lowering A.

References

1. Cooper, R. L., Conn, P. M. & Walker, R. F. Characterization of the LH surge in middle-aged female rats. *Biol. Reprod.* **23**(3), 611–615; 10.1095/biolreprod23.3.611 (1980).

2. Wise, P. M. et al. Neuroendocrine influences and repercussions of the menopause. *Endocr. Rev.* **20**(3), 243–248; 10.1210/edrv.20.3.0364 (1999).

3. Takasu, N. N. et al. Recovery from age-related infertility under environmental light-dark cycles adjusted to the intrinsic circadian period. *Cell Rep.* **12**(9), 1407–1413; 10.1016/j.celrep.2015.07.049 (2015).

4. Wittmann, M., Dinich, J., Merrow, M. & Roenneberg, T. Social jetlag: misalignment of biological and social time. *Chronobiol. Int.* **23**(1-2), 497–509; 10.1080/07420520500545979 (2006).

5. Roenneberg, T., Allebrandt, K. V., Merrow, M. & Vetter, C. Social jetlag and obesity. *Curr. Biol.* **22**(10), 939–943; 10.1016/j.cub.2012.03.038 (2012).

6. Kuramoto, Y. Chemical Oscillations, Waves, and Turbulence (Springer, Berlin); 10.1007/978-3-642-69689-3 (1984).

7. Carmichael, M. S., Nelson, R. J. & Zucker, I. Hamster activity and estrous cycles: control by a single versus multiple circadian oscillator(s). *Proc. Natl. Acad. Sci. USA* **78**(12), 7830–7834;

10.1073/pnas.78.12.7830 (1981).

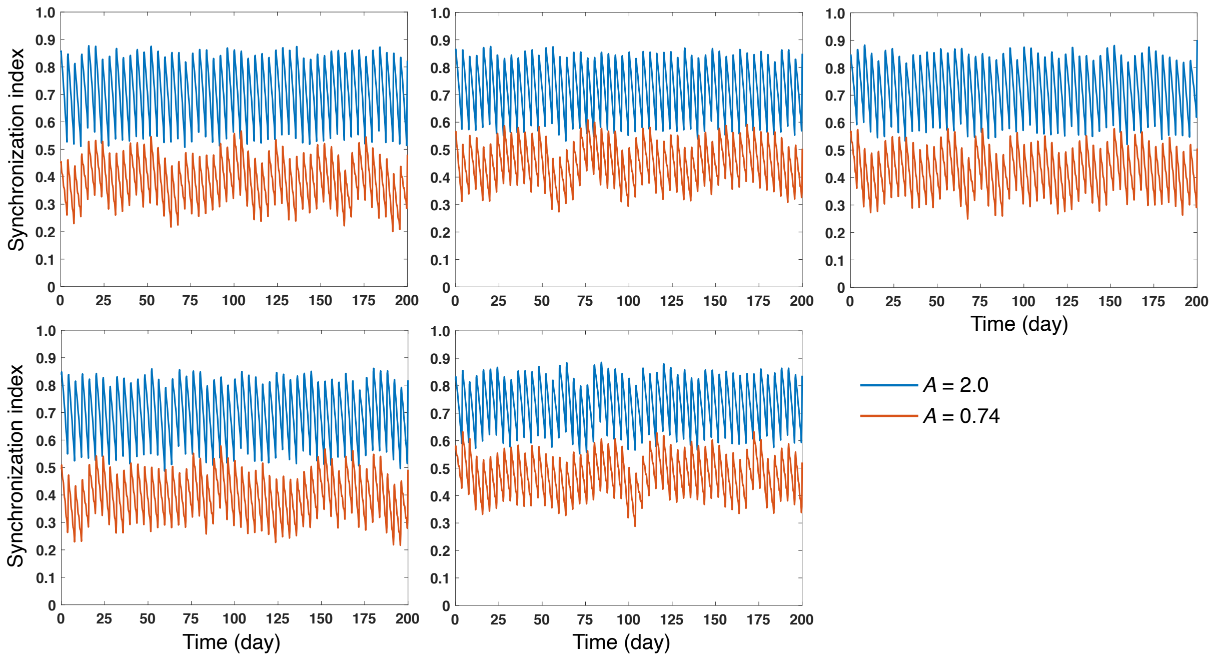

Supplementary Figure S1. Timeseries of synchronization indices for large ($A = 2.0$) and small ($A = 0.74$) amplitudes of SCN signal obtained from five distinct simulations with different sets of randomly chosen ovarian circadian periods. An upper left panel corresponds to the data mainly discussed in the text.

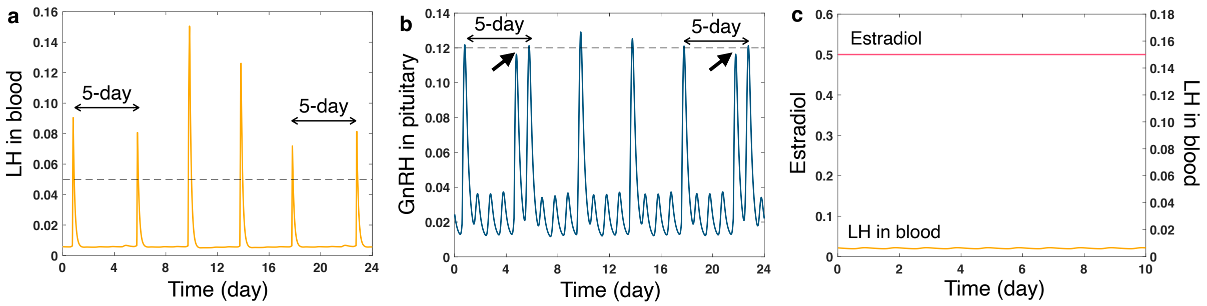

Supplementary Figure S2. Profiles of hormone levels with very small amplitude of SCN signal (A = 0.66 in (**a**) and (**b**), and A = 0.65 in (**c**)). (**a**) LH in blood. (**b**) GnRH in pituitary. Black arrows represent subthreshold increase of GnRH. (**c**) Estradiol (left axis) and LH in blood (right axis). Dashed lines in (**a**) and (**b**) stand for threshold values (G^* and L^*, respectively). Each timeseries is a snapshot extracted arbitrarily from the long-term simulation data. The simulation time does not indicate age of the animal (see also Simulation conditions in Supplementary Methods for interpretation of time).

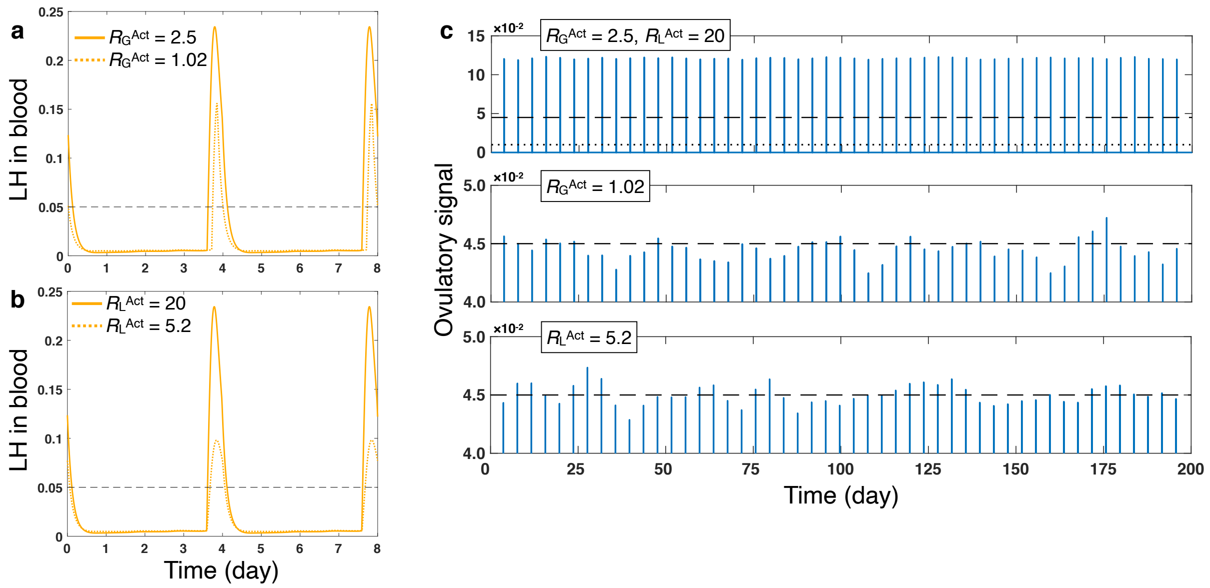

Supplementary Figure S3. Dynamics of (**a**, **b**) LS and (**c**) ovulation for different values of $R_{\mathrm{G}}^{\mathrm{Act}}$, activation effect of estradiol on GnRH release, and $R_{\mathrm{L}}^{\mathrm{Act}}$, activation effect of GnRH on LH release. $R_{\mathrm{G}}^{\mathrm{Act}} = 2.5$ and $R_{\mathrm{L}}^{\mathrm{Act}} = 20$ are default values of these parameters. Dashed lines in (**a**, **b**) stand for a threshold L^*. Dashed and dotted lines in (**c**) represent high and low values of ovulation threshold P^*, respectively. Each timeseries is a snapshot extracted arbitrarily from the long-term simulation data. The simulation time does not indicate age of the animal (see also Simulation conditions in Supplementary Methods for interpretation of time).

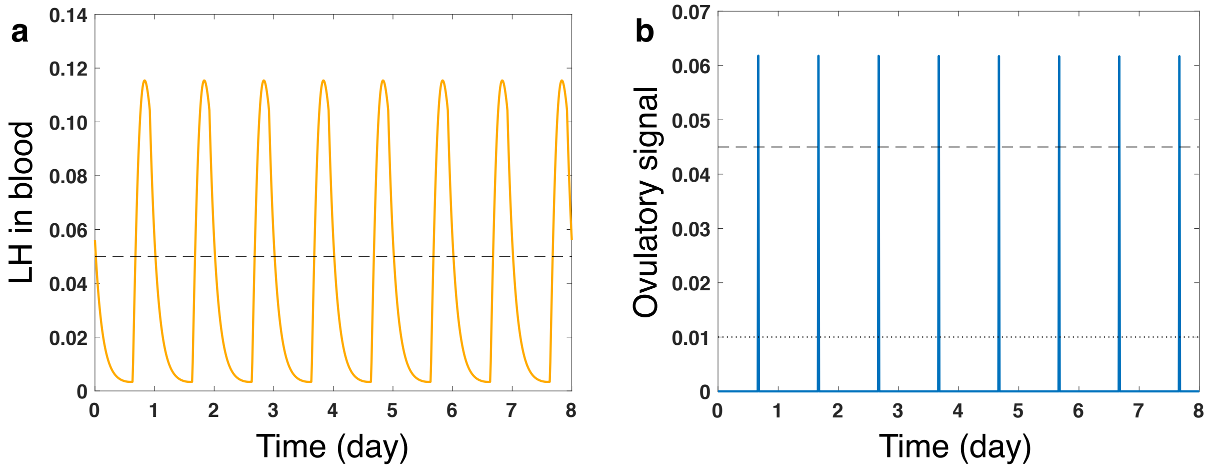

Supplementary Figure S4. Abnormal reproductive cycles caused by very large amplitude of SCN signal (A = 10). (**a**) LH in blood. (**b**) Ovulatory signal. A dashed line in (**a**) stand for threshold L^*, and dashed and dotted lines in (**b**) stand for high and low ovulation threshold P^*, respectively.

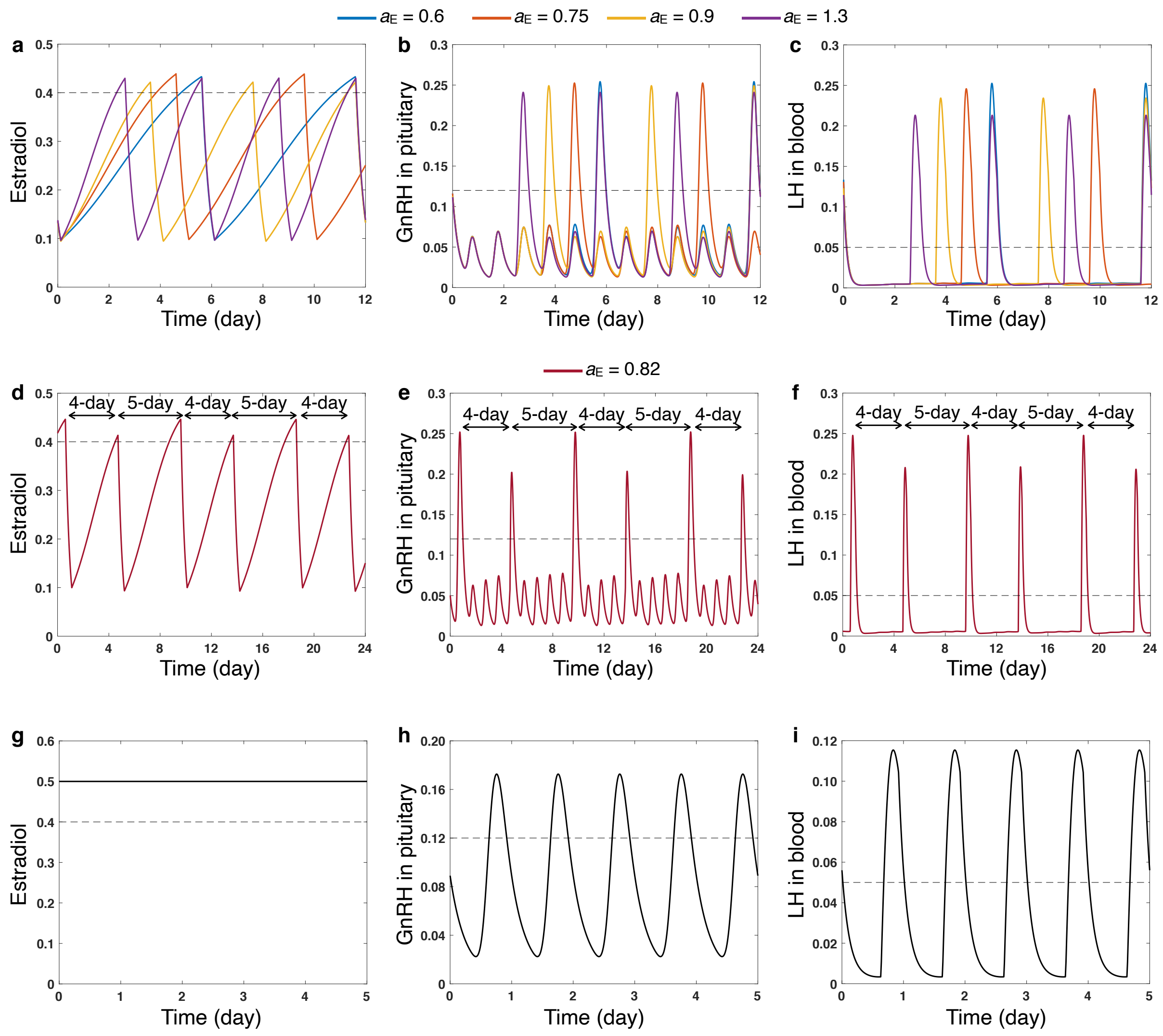

Supplementary Figure S5. Hormone dynamics for (**a**–**f**) different values of a_E (synthesis rate of estradiol) and with (**g**–**i**) assumption of constant estradiol levels. (**a**, **d**, **g**) Estradiol. (**b**, **e**, **h**) GnRH in pituitary. (**c**, **f**, **i**) LH in blood. In (**c**) regular LS with 6-, 5-, 4- and 3-day intervals is induced when a_E is set to values of 0.6, 0.75, 0.9 (default value), and 1.3, respectively. Dashed lines in (**a**, **d**, **g**), (**b**, **e**, **h**), and (**c**, **f**, **i**) stand for threshold values (E^*, G^* and L^*, respectively). Each timeseries is a snapshot extracted arbitrarily from the long-term simulation data. The simulation time does not indicate age of the animal (see also Simulation conditions in Supplementary Methods for interpretation of time).

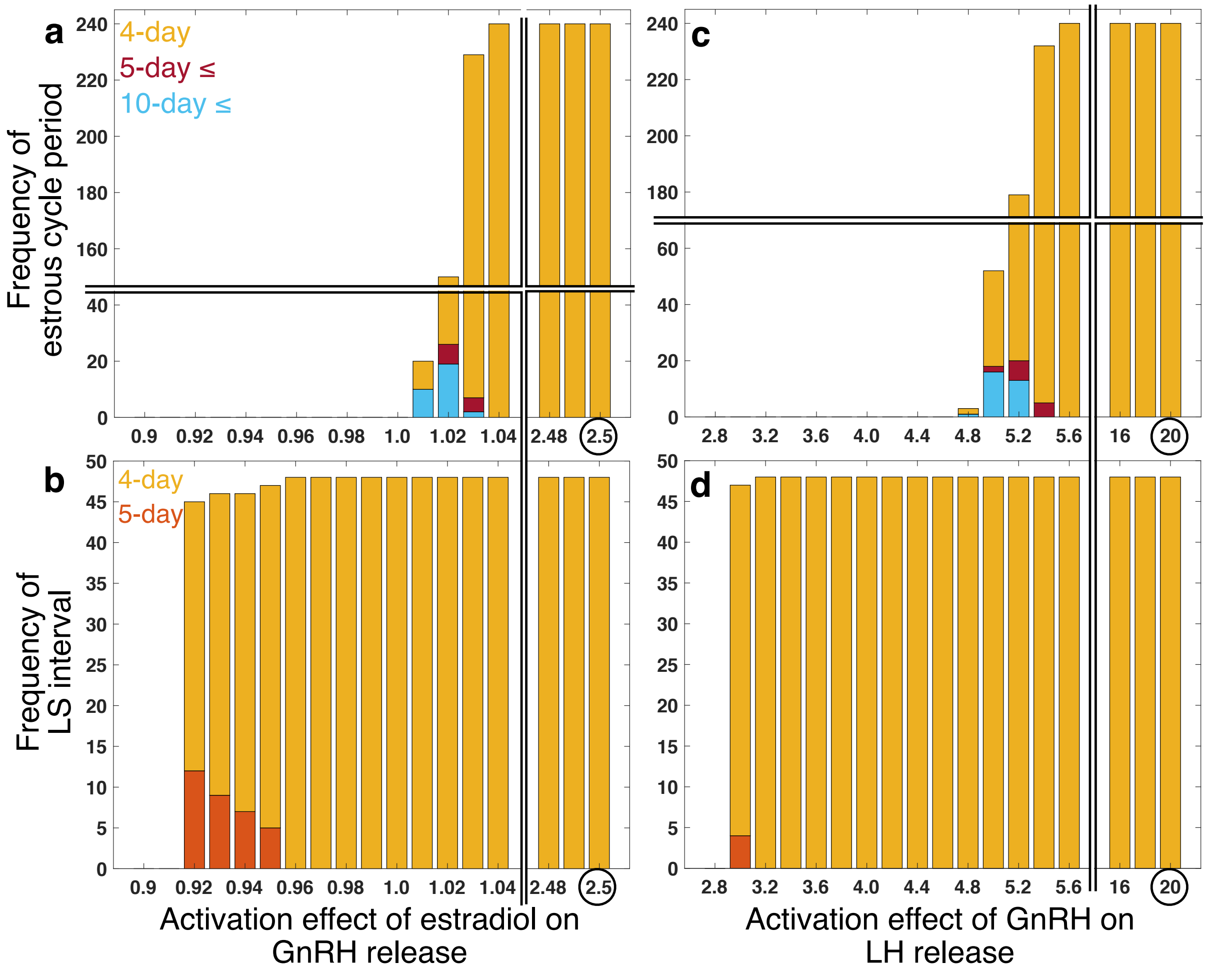

Supplementary Figure S6. Estrous cycle period and LS interval depend on (**a**, **b**) activation effect of estradiol on GnRH release and (**c**, **d**) activation effect of GnRH on LH release. (**a**, **c**) Frequency of estrous cycle periods for different parameter values. Ovulation threshold P^* is set to $4.5 * 10^{-2}$. Data obtained from five trials, which are based on distinct realization of randomly-chosen periods of ovarian circadian clocks, are lumped together and used to depict the graphs. (**b**, **d**) Frequency of LS intervals for different parameter values. Circles indicate default parameter values. In (**a**) and (**b**), the estrous cycle period and the LS interval, respectively, are always 4 days between parameter values of 1.04 and 2.48. In (**c**) and (**d**), the estrous cycle period and the LS interval, respectively, are always 4 days between parameter values of 5.6 and 16.

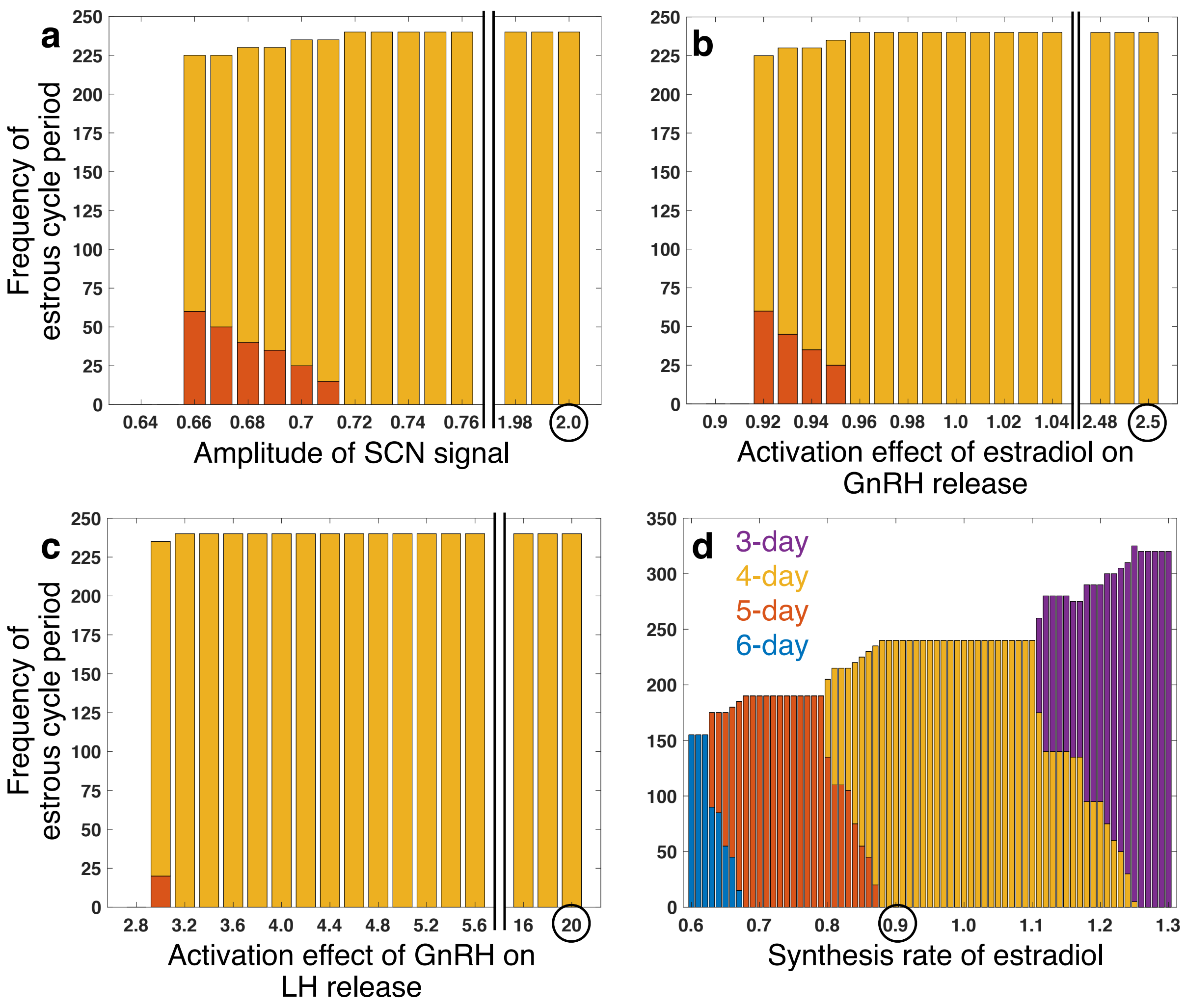

Supplementary Figure S7. Dependence of estrous cycle period for low ovulation threshold ($P^* = 1.0 * 10^{-2}$) on (**a**) amplitude of SCN signal, (**b**) activation effect of estradiol on GnRH release, (**c**) activation effect of GnRH on LH release, and (**d**) synthesis rate of estradiol. Circles in each graph indicate default parameter values. Data obtained from five trials, which are based on distinct realization of randomly-chosen periods of ovarian circadian clocks, are lumped together and used to depict the graphs. White bars in (**a**–**c**) indicate that the estrous cycle period is always 4 days between two parameter values. Color codes for different periods are shown in (**d**).

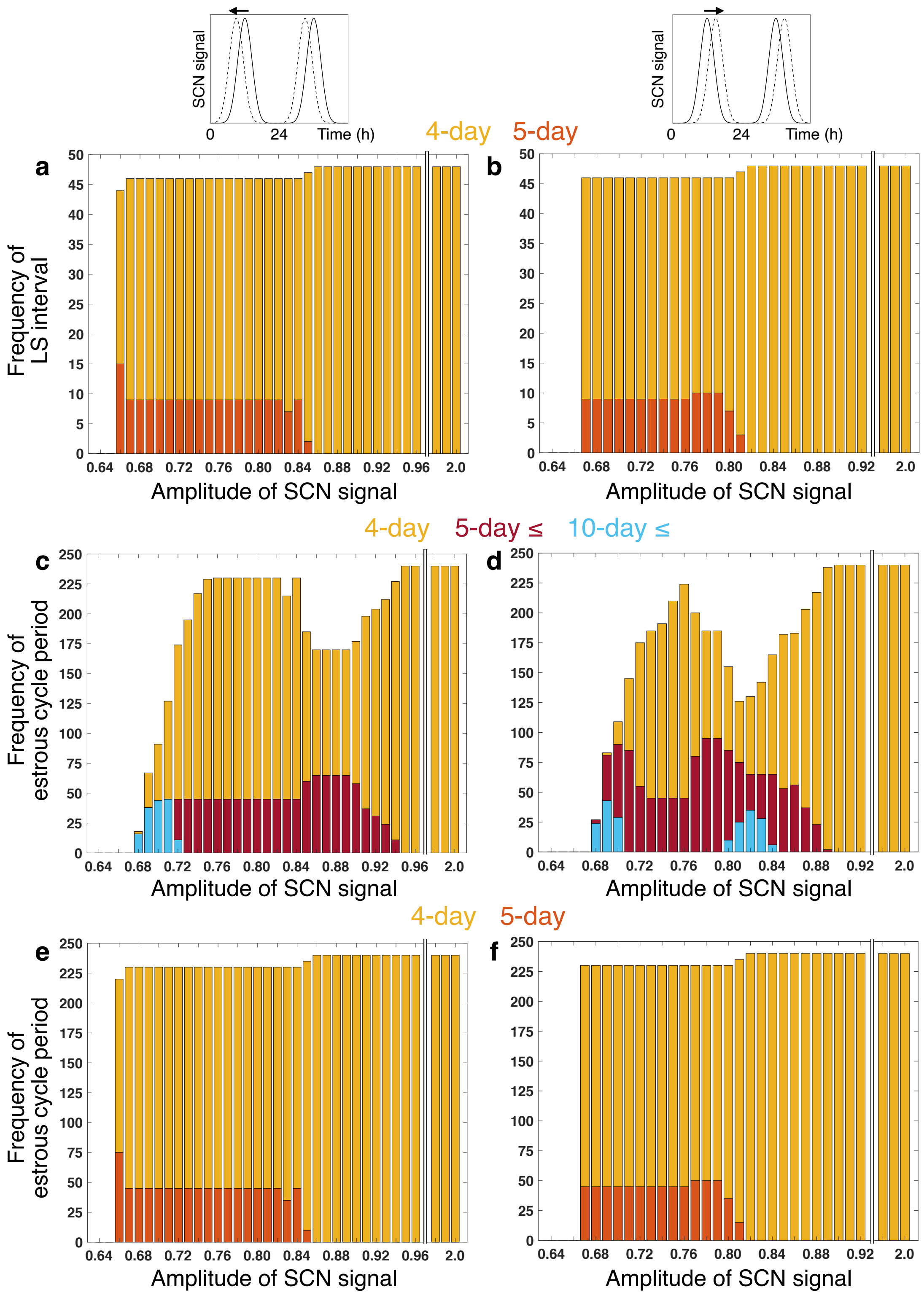

Supplementary Figure S8. Frequency of (**a**, **b**) LS interval and (**c**–**f**) estrous cycle period in animals perturbed by variation of phase of SCN signal. Perturbations are the repeated 3-hour advance in the phase of the SCN signal in (**a**), (**c**), and (**e**), while the repeated 3-hour delay in (**b**), (**d**), and (**f**). The LS interval in (**a**) and the estrous cycle period in (**c**) and (**e**) are always 4 days between parameter values of 0.96 and 1.98. The LS interval in (**b**) and the estrous cycle

period in (**d**) and (**f**) are always 4 days between parameter values of 0.92 and 1.98. Ovulation threshold P^* is set to 4.5 * 10^{-2} in (**c**) and (**d**) and to 1.0 * 10^{-2} in (**e**) and (**f**). Data obtained from five trials, which are based on distinct realization of randomly-chosen periods of ovarian circadian clocks, are lumped together and used to depict the graphs.

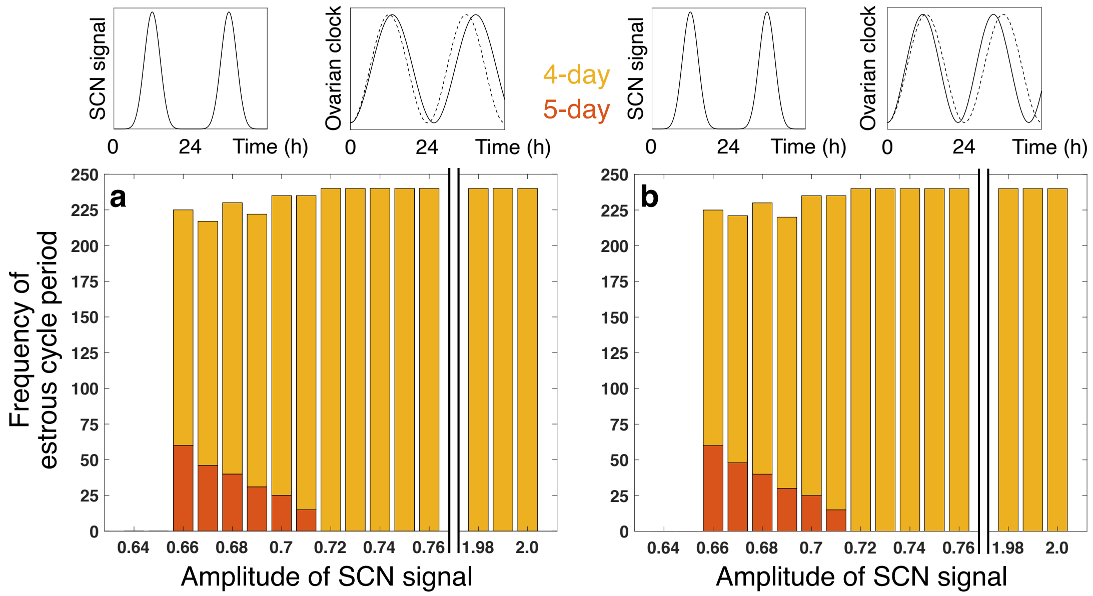

Supplementary Figure S9. Frequency of estrous cycle period for various SCN signal amplitudes in (**a**) O-long and (**b**) O-short mutants with low P^* ($1.0 * 10^{-2}$). In (**a**) and (**b**) the estrous cycle period is always 4 days between parameter values of 0.76 and 1.98. Data obtained from five trials, which are based on distinct realization of randomly-chosen periods of ovarian circadian clocks, are lumped together and used to depict the graphs.

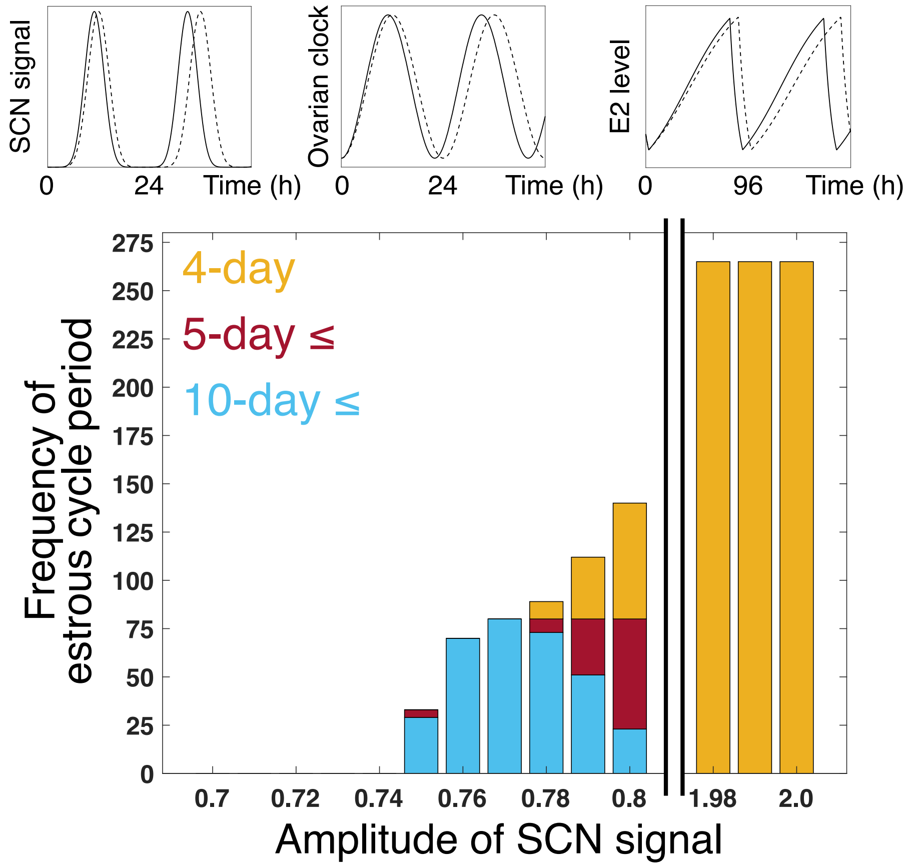

Supplementary Figure S10. Adjustable estradiol synthesis rate affects estrous cyclicity of O-short mutant under short environmental cycle. Frequency of estrous cycle period for various SCN signal amplitude is shown. Ovulation threshold P^* is set to $4.5 * 10^{-2}$. The estrous cycle period is always 4 days between parameter values of 0.8 and 1.98. Data obtained from five trials, which are based on distinct realization of randomly-chosen periods of ovarian circadian clocks, are lumped together and used to depict the graphs.

Supplementary Table S1

Summary of variables, functions, and parameters.

		Definition	Quantity	Value
Variables	G_{Hypo}	GnRH level in the hypothalamus		$G_{\mathrm{Hypo}}(0) = 1.0$
	G_{Pit}	GnRH level in the pituitary		$G_{\mathrm{Pit}}(0) = 0.01$
	L_{Pit}	LH level in the pituitary		$L_{\mathrm{Pit}}(0) = 1.0$
	L_{Blood}	LH level in the blood		$L_{\mathrm{Blood}}(0) = 0.01$
	$E2$	Estradiol level in the ovary		$E2(0) = 0.1$
	φ_j	Phase of jth ovarian circadian oscillator	rad	$\varphi_j(0) = 0$
Functions	C	Hypothalamic circadian signal		
	R_{G}	$E2$-dependent release rate of G_{Hypo}	1/hour	
	R_{L}	G_{Pit}-dependent release rate of L_{Pit}	1/hour	
	P_i	Ovulatory signal resulting from the ith LS event		
	S_j	Sensitivity to LH of the jth ovarian circadian oscillator		
	S	Sensitivity to LH of the ovary as a whole		$(1/N)\sum_{j=1}^{N} S_j$
	Z_j	Phase response curve to LH stimulus of the jth ovarian circadian oscillator	rad/hour	
Parameters	a_{G}	Synthesis rate of G_{Hypo}	$[G_{\mathrm{Hypo}}]$/hour	0.02083
	a_{L}	Synthesis rate of L_{Pit}	$[L_{\mathrm{Pit}}]$/hour	0.02083
	a_{E}	Synthesis rate of $E2$	1/hour	0.0375
	G_{Max}	Maximum level of G_{Hypo}	$[G_{\mathrm{Hypo}}]$	1.0
	L_{Max}	Maximum level of L_{Pit}	$[L_{\mathrm{Pit}}]$	1.0
	E_{Max}	Maximum level of $E2$	$[E2]$	0.5
	r_{G}	Basal release rate of G_{Hypo}	1/hour	0.002083
	r_{L}	Basal release rate of L_{Pit}	1/hour	0.002083
	$R_{\mathrm{G}}^{\mathrm{Act}}$	$E2$-dependent activation effect on G_{Hypo} release	1/hour	2.5
	$R_{\mathrm{G}}^{\mathrm{Rep}}$	$E2$-dependent repression effect on G_{Hypo} release	1/hour	0.5
	$R_{\mathrm{L}}^{\mathrm{Act}}$	G_{Pit}-dependent activation effect on L_{Pit} release	1/hour	20
	$R_{\mathrm{L}}^{\mathrm{Rep}}$	G_{Pit}-dependent repression effect on L_{Pit} release	1/hour	0.1

	c_G	Clearance rate of G_{Pit}	1/hour	0.167
	c_L	Clearance rate of L_{Blood}	1/hour	0.333
	c_E	Clearance rate of *E2*	1/hour	0.125
	G^*	Threshold value of G_{Pit} for GS	$[G_{Pit}]$	0.12
	L^*	Threshold value of L_{Blood} for LS	$[L_{Blood}]$	0.05
	E^*	Threshold value of *E2* for ES	[*E2*]	0.4
	τ_E	Time delay of *E2* feedback effect on G_{Hypo} release	hour	4
	t_i^{On}	The onset time of the *i*th LS event	hour	
	τ_{LS}	Duration of the LS event	hour	12
	T	Period of environmental cycles	hour	24 (Normal cycle)<br>22 (Short cycle)<br>26 (Long cycle)
	A	Amplitude of C		2.0
	ω_C	Natural frequency of C	rad/hour	$2\pi/T$
	n	Constant determining shape of C		5
	a_C	Constant determining shape of C		0.5
	b_C	Constant determining shape of C		0.45
	ψ_C	Phase of C	hour	10
	v_C	Scaling factor for ψ_C		$T/24$
	N	Number of ovarian cellular oscillators		200
	τ_j	Free-running period (FRP) of the *j*th ovarian circadian oscillator	hour	
	τ_O	Mean of FRP of ovarian circadian oscillators	hour	22 (O-short mutant)<br>26 (O-long mutant)<br>24 (Other genotypes)
	ω_j	Natural frequency of the *j*th ovarian circadian oscillator	rad/hour	$2\pi/\tau_j$
	γ	Constant determining the strength of LH stimulus	$1/[L_{Blood}]$	20
	ψ_Z	Phase of Z_j	hour	2
	v_Z	Scaling factor for ψ_Z		$\tau_j/24$
	P^*	Threshold value of P_i for ovulation		0.045 or 0.01
	τ_P	Duration during which P_i is calculated	hour	12
	a_S	Mean of S_j		1

	b_S	Amplitude of S_j		1
	ψ_S	Phase of S_j	hour	8
	v_S	Scaling factor for ψ_S		$\tau_j/24$

Supplementary Table S2

The values of the SCN signal amplitude *A*, below which the multi-periodicity and the arrest of estrous cycle are observed.

Genotype	Ovulation threshold P^*	Period of the SCN signal T (h)	Multi-periodicity of estrous cycle	Arrest of estrous cycle
Wild type	$4.5 * 10^{-2}$	24	A = 0.75 (Fig. 4a)	A = 0.69 (Fig. 4a)
		24 with repeated phase advance	A = 0.95 (Fig. S8c)	A = 0.68 (Fig. S8c)
		24 with repeated phase delay	A = 0.90 (Fig. S8d)	A = 0.68 (Fig. S8d)
	$1.0 * 10^{-2}$	24	A = 0.72 (Fig. S7a)	A = 0.66 (Fig. S7a)
		24 with repeated phase advance	A = 0.86 (Fig. S8e)	A = 0.66 (Fig. S8e)
		24 with repeated phase delay	A = 0.82 (Fig. S8f)	A = 0.67 (Fig. S8f)
O-long	$4.5 * 10^{-2}$	24	A = 0.84 (Fig. 6a)	A = 0.77 (Fig. 6a)
		26	A = 0.65 (Fig. 6c)	A = 0.63 (Fig. 6c)
	$1.0 * 10^{-2}$	24	A = 0.72 (Fig. S9a)	A = 0.66 (Fig. S9a)
O-short	$4.5 * 10^{-2}$	24	A = 0.86 (Fig. 6b)	A = 0.78 (Fig. 6b)
		22	A = 2.0 (Fig. 6d)	A = 0.72 (Fig. 6d)
	$1.0 * 10^{-2}$	24	A = 0.72 (Fig. S9b)	A = 0.66 (Fig. S9b)
